# Control of pest ants by pathogenic fungi: state of the art

**DOI:** 10.3389/ffunb.2023.1199110

**Published:** 2023-10-11

**Authors:** Patricia J. Folgarait, Daniela Goffré

**Affiliations:** Ants Laboratory, Department of Science and Technology, Quilmes National University, National Scientific and Technical Research Council (CONICET), Bernal, Buenos Aires, Argentina

**Keywords:** ant mortality, biocontrol, invasive ants, entomopathogens, mycopathogens, fields studies, laboratory studies, strains efficacy

## Abstract

Pest ants are known for their damage to biodiversity, harm to agriculture, and negative impact on human welfare. Ants thrive when environmental opportunities arise, becoming pests and/or invading non-native areas. As social insects, they are extremely difficult to control using sustainable methods like biological control. The latter, although safer to the environment, acts slowly allowing the ants to use their individual and social defenses. Among biocontrol agents, fungal pathogens were proposed as promising, however, it is difficult to ascertain their success when the bibliography has not been reviewed and condensed. Therefore, this paper is the first in performing such task by analyzing publications mainly from 2000 to 2022 about the control of pest ants by fungi. From 85 publications selected, 77% corresponded to laboratory studies. *Beauveria* and *Metarhizium* were the genera most used in laboratory and field studies. Most of them included *Acromyrmex* and *Atta* leaf-cutter ants (LCA), and *Solenopsis* fire ants. From laboratory experiments, we evaluated how ant net mortality was affected by ant and fungal species, and also by origin, concentration, and inoculation technique of the fungal strains tested. *Beauveria bassiana* and *Metarhizium anisopliae* produced the greatest mortality, along with the inoculation spray technique and fungal strains collected from ants. There was a positive relationship between ant mortality and fungal concentration only for those studies which evaluated more than one concentration. Twenty field experimental studies were found, covering 13 pest species, mainly LCA and *Solenopsis invicta*. Only *B. bassiana* was tested on *Solenopsis*, *M. anisopliae* was mostly used for *Acromyrmex*, and *M. anisopliae* or *Trichoderma* were mainly used with *Atta* species. The median control field efficiency varied from 20% to 85% for different fungi and ant genera. When grouping all fungal species together, the median control efficiency seemed to be better for *Acromyrmex* (67%) than for *Atta* and *Solenopsis* (both 43%). Our review shows that, at this stage of knowledge, it is very difficult to extrapolate any result. We offer suggestions to improve and standardize laboratory and field experimental studies in order to advance more efficiently in the fungal control of pest ants.

## 1 Introduction

Biological control is an ancient strategy (Doutt, 1964), by which one species is used to control the population of another or to decrease the impact that the pest produces (Eilenberg et al., 2001). In general, the mechanism of action is through predation or parasitism by pathogens, predators, or parasitoids. Biological control has been preferred over traditional methods of control, such as the use of synthetic pesticides, because it is more specific, non-contaminant, does not accumulate through food chains, and is safe for applicators (Goettel et al., 1990; Zimmermann, 1993; Goettel et al., 2001; Vestergaard et al., 2002; Taylor and Snyder, 2021). Biological control of insects has been employed intensively since the 20^th^ century (Burges, 1981; Johnson and Stockwell, 2000; see more references in Lacey et al., 2015), and several biopesticides have been launched to the market (Kabaluk and Gazdik, 2005; Borgio and Sahayaraj, 2011; Lacey et al., 2015), however, this is not the case for ant pests.

Ants are one of the most abundant organisms in the world (Schultheiss et al., 2022). They occupy any habitat except those at very high altitudes or extremely cold. They have been considered ecosystem engineers and have many beneficial effects on soil and plants (Folgarait, 1998). As social insects, they have enormous population growth potential and overlapping generations, therefore, if the conditions are appropriate, they tend to exponentially increase their numbers producing several problems to human welfare and the economy. The biological control of pest ants started with the search of natural enemies for the control of invasive fire ants in the US (Williams et al., 1973; Allen and Buren, 1974) after the government invested billions of dollars in unsuccessful chemical control (Williams et al., 2001). Later, and mainly in the 90s, studies of biological control began with the leaf-cutter ants (LCA) (Diehl-Fleig et al., 1993; Jaccoud et al., 1999). It is surprising to note that very little has been done on this topic compared to the much greater amount of work performed to control other insects (DeBach, 1964; Fisher et al., 1999). Most probably, the reasons are related to the challenge that social insects impose to achieve an efficient control. Ants have several lines of defense, from behavioral and chemical, to immunological, at the individual level. Among the behavioral defenses, the most common are related to grooming, where ants remove pathogens mechanically and/or kill them by producing compounds that are spread through their bodies (Obin and VanderMeer, 1985; Oi and Pereira, 1993; Phair, 2020; Roy et al., 2006; Richard and Errard, 2009; Pull et al., 2018; Tragust et al., 2013; Goes et al., 2022). In the case of parasitoids, ants attempt to catch, chase, and/or spray venoms to avoid being parasitized (Wuellner et al., 2002; Elizalde and Folgarait, 2012). Another very common defense strategy in ants is the production of antimicrobial compounds from their metapleural glands (Fernández-Marín et al., 2006; Little et al., 2006; Yek and Mueller, 2011). In the case of many *Acromyrmex* LCA, they also harbor actinomycete bacteria on their exoskeleton, which produce antifungals (Currie et al., 1999). In addition, many ants use their infrabuccal pocket to filter out small particles, such as bacterial and fungal spores, presumably to avoid infections (Glancey et al., 1981; Little et al., 2003).

Fungi are one of the most important threats for soil ants because this environment commonly contains this type of organism (Lacey et al., 2015), which may be pathogenic to ants (Keller and Zimmermann, 1989). In addition to the defenses at the individual level explained above, ants exhibit social defenses performed at colony level towards this type of pathogen (Cremer et al., 2007). It has been shown that ants can change their sanitary behaviors depending on the type of fungi that attacks them (Walker and Hughes, 2009; Goes et al., 2022). In fact, great behavioral plasticity has been demonstrated in weakly infected ants to avoid their own superinfection with a second harmful pathogen, by reducing allogrooming (grooming another ant) and increasing the application of antifungals towards contaminated nestmates (Ben-Ami et al., 2011). It has also been shown that a non-mortal fungal infection offers greater resistance to future exposures (priming immunization, Masri and Cremer, 2014) and, most interestingly, it can promote immunization among non-infected nestmates through the social transfer of the fungal pathogen (Konrad et al., 2012). Relative only to LCA, these ants include infections-avoidance behaviors in their fungal garden, such as weeding, to remove alien fungal conidia and infected pieces of mycelia (Currie and Stuart, 2001). In addition, the contaminants are removed outside the nests by using a special worker caste to reduce contact and thus the spread of infected material (Hart and Ratnieks, 2001). Therefore, the control of pest ants has to circumvent all or most of the individual and social defenses ants have, making it particularly challenging.

### 1.1 Characteristics of invasive and pest ant species

Most of the ant pest species known are also invasive exotic species. Exotic species are those that reach a non-native, far away region, and become invasive when they establish there and can spread within the introduced range, become dominant and, in turn, negatively affect local biodiversity and human welfare (Ricciardi et al., 2000). This type of species is typically found on islands, such as Hawaii (Reimer, 1994) and the Galapagos archipelago (Brandão and Paiva, 1994). However, several ant species have invaded new areas from continents far away from native ones. The most known, and most studied, exotic invasive species are the red fire ants (*Solenopsis invicta*) which have invaded the US, Mexico, Australia, China, Taiwan, Korea, Japan (Callcott and Collins, 1996; Moloney and Vanderwoude, 2002; Sanchez-Peña et al., 2009; Wetterer, 2013; Wang and Lu, 2017), and the Argentine ants (*Linepithema humile*) which invaded the US, part of Europe, Australia, New Zealand, and Korea (Van Schlagen et al., 1994; Suarez et al., 2001; Giraud et al., 2002; Wild, 2004; Ward et al., 2005; Lee et al., 2020). There are many other invasive ant species, comparatively less studied but also considered pests either due to their economic impact or their negative effect on the biodiversity of the invaded community and/or public health (Williams, 1994; Wetterer and Porter, 2003; Wang et al., 2016; Lee et al., 2020; Xu et al., 2022). Among the reasons why invasive ant species become pests in exotic places is the fact that they leave their natural enemies behind (Porter et al., 1997; Torchin et al., 2003; Hajek and Eilenberg, 2018), although lately it has been argued that invasive ants act as a disease reservoir (Cremer, 2019). According to Williamson (1996), 10% of the hundreds of species that are recorded in exotic places can establish in new areas. Most probably, this percentage is lower for ants as these insects require the dispersion of a mated queen (few invasive ants have asexual reproduction; Eyer and Vargo, 2021). However, if mated queens were to reach exotic places, and the abiotic conditions were favorable, the chances for a successful establishment would increase. Furthermore, if the exotic ant species has the traits of an invasive syndrome, then their probability of establishing and becoming dominant increases (Novoa et al., 2020). In the case of exotic, invasive ants, those traits have been clearly identified in the literature (Passera, 1994; Eyer and Vargo, 2021): polygyny (several queens per colony; Haines and Haines, 1978; Keller, 1988), unicoloniality (due to a relaxed territoriality and discrimination among nestmates; Markin, 1968), presence of polydomy (several nests for one colony; Edwards, 1986), opportunistic and omnivorous habits (Passera, 1994), intranidal mating and colony foundation by budding (queens moving by foot from maternal colony followed by workers; Peacock et al., 1955; Ulloa-Chacon, 1990), and high levels of aggression in competitive interspecific encounters (Fowler, 1993; Holway et al., 2002; Le Brun et al., 2007). Tramp ant species follow humans’ movements and they mostly live in close association with humans in urban areas and could be considered a type of invasive species (Passera, 1994).

There are other ant pests that are “invasive” species but not exotic. This group refers to those ants that generate an economic loss to humans by increasing their numbers significantly in disturbed habitats or urban areas of their native range. This is the emblematic case of LCA, which are one of the most damaging ant species in the Neotropics, taking advantage of monocultures, generating enormous economic impacts by reducing crop/plantations yields (Della Lucia, 1993; Jiménez et al., 2021). Most probably, there are other ant species in this group besides LCA, although they are hardly considered or studied. For example, *Camponotus punctulatus* ants live under the soil in the natural grasslands of Argentina, but soil disturbance (e.g. cattle pastures or rice agriculture) generates a change in their lifestyle, becoming epigeal and exploding demographically (Folgarait et al., 2007). Their above-ground nests become enormous and extremely hard to break, decreasing the land value and significantly increasing farmers’ expenses when trying to break them in order to prepare the soil for cultivation. There are not enough studies to date to confirm or reject if this group of pest ants shares the same trait syndrome that characterizes exotic and tramp species. However, crazy ants (*Nylanderia fulva* which are also exotic invaders, Kumar et al., 2015) and Argentine ants become pests in urban areas and/or invasive within their native range, becoming a nuisance for people and a health threat in hospitals (Josens et al., 2014).

### 1.2 Fungi as biological control agents

Ant pests have been traditionally controlled by chemical pesticides and most of the information available is related to this method of control (for example, see de Britto et al., 2016). However, the biological control of ants has been growing lately (this study shows a 170% increase in the number of publications about that topic from 2000 to 2022, see **Table 1**), most probably as a consequence of the negative effects of the chemical methods and the new rules, demands, and certifications requested to farmers and foresters (Forest Stewardship Council, 2019; Stockholm Convention on persistent organic pollutants, 2019).

**Table 1:** Number of publications for the period 2000-2022, discriminated by year.

Year	AR	WR	I
2000	84	0	0
2001	92	2	1
2002	58	5	2
2003	38	5	1
2004	60	3	2
2005	82	2	1
2006	56	5	4
2007	72	5	1
2008	96	3	0
2009	99	5	0
2010	126	7	3
2011	115	10	8
2012	138	15	6
2013	158	10	0
2014	129	19	3
2015	134	15	4
2016	146	16	5
2017	159	19	7
2018	167	18	6
2019	161	16	7
2020	187	23	7
2021	175	17	5
2022	145	17	2
**Total**	***2677***	***237***	***75***

AR = number of abstracts read to evaluate the scope of each publication; WR = number of papers read in full; I = number of publications included in this study for the mentioned period.

Among organisms that can control ants, pathogenic fungi are the predominant natural pathogens in arthropod populations and possess many desirable traits that favor their use as biological control agents (Lacey and Goettel, 1995; Lacey et al., 2015). Those traits include the possibility to cause epizootics (an outbreak of the disease affecting many host insects of the same kind at the same time), indicating their potential for the reduction of pest populations, and, at the same time, to lower the risk to beneficial organisms (Goettel et al., 2001; Traugott et al., 2005; Brownbridge and Glare, 2007; O’Callaghan and Brownbridge, 2009; Goffré and Folgarait, 2019; Folgarait and Goffré, 2021a), which can also contribute to regulating pest populations (Lacey et al., 2015; Hajek and Eilenberg, 2018). In addition, fungal mass-production systems have been developed to provide large quantities of inoculum, which can then be formulated and repeatedly applied as sprays, granules, etc. (Shah and Pell, 2003; Lacey et al., 2015). Furthermore, unlike viruses and many nematodes and bacteria, which require specialized routes of entry for infection of insect hosts, entomopathogenic fungi infect via penetration of the host cuticle anywhere (St Leger et al., 1986; Hajek and St. Leger, 1994; Ortiz-Urquiza and Keyhani, 2013).

The first step of the pathogenic process is to adhere the conidia to the insect cuticle. Afterwards, using a variety of hydrolytic enzymes, e.g. proteases, chitinases, and lipases, and forming structures such as germ tubes, appressoria and/or penetration pegs, the fungus breaches the host integument reaching the insect haemocele (Shah and Pell, 2003; Ortiz-Urquiza and Keyhani, 2013). After that, the fungus needs to circumvent the host’s immunological defenses by growing its mycelia extremely fast and/or producing toxins to kill the host (Charnley, 2003; Schrank and Vainstein, 2010). Infection and host death occur through different pathways in relation to the group of fungi: Hyphomycetes can be hemibiotrophic with a well-defined parasitic phase within the alive host, plus a later saprophytic phase after the insect dies; whereas Entomophthorales have biotrophic relationships with little or no saprophytism (which could accelerate the time of death) (Shah and Pell, 2003). Therefore, insect control by entomopathogenic fungi is achieved when sufficient infective propagules (generally conidia) contact a susceptible host and conditions are suitable to develop a lethal mycosis (Lacey et al., 2015).

In addition, mycoparasites are another important strategy in the control of LCA, as an indirect way of controlling the entire colony by reducing their main source of food (i.e. its symbiotic fungi). The most studied fungal genera with mycotrophic characteristics are *Escovopsis* and *Trichoderma* (Marfetán et al., 2018; Cotazo-Calambas et al., 2022). These and other microorganisms access the fungal gardens and become a threat to the entire colony if they become abundant (Jiménez-Gómez et al., 2021). The simultaneous use of two types of pathogens acting on different targets (ants and cultivated fungus) should be a better strategy for the control of LCA (Lopez and Orduz, 2003; Fernandez-Daza et al., 2019; Folgarait and Goffré, 2021b).

Despite the importance of fungi as biological control agents (Lacey et al., 2015), no systematic search of the literature has been done about the fungal control of pest ants (exotic or not). Therefore, a critical review of this topic is needed to summarize findings, identify knowledge gaps, and provide recommendations for future research. In turn, those are the goals of this study. We focused on laboratory experiments, where most of the work has been done, evaluating the effect of fungal species and strains on ant mortality, in addition to other variables reported. Nevertheless, we put an emphasis on field studies as these are the ones that can truly evaluate the effect of the biological control proposed.

## 2 Methods

### 2.1 Publications selection

**Figure 1:**
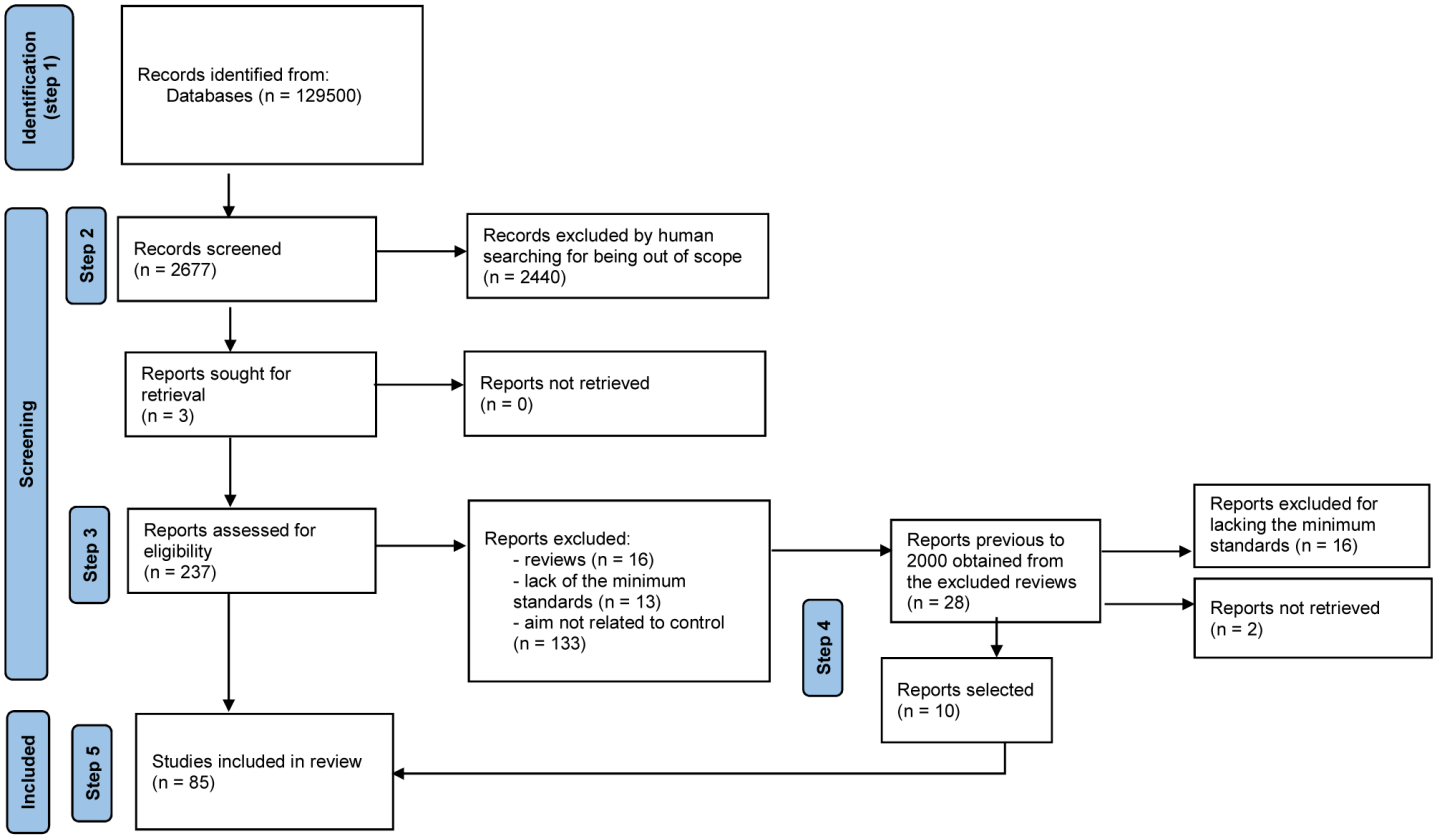
Flow diagram showing the steps followed to select the publications used in this study, starting from a systematic reviews of publications from 2000 to 2022, using Ants+”Biological control”+Fungi as keywords for the searching.

A search of the literature available was performed using Google Scholar with the keywords Ants+”Biological control”+Fungi (words enclosed in quotation marks were considered as one by the browser) (**Figure 1**). Because the number of pages was >16,000, we decided to restrict the search by year considering each year from the interval 2000-2022. We used the “time interval” filter tool to search for citations by year, which summed up 12,950 pages (see number of publications by year in **Table 1**) for the 22-years period. In order to select among the citations, we followed several steps. The first step involved looking at the titles and keywords rendered by the search to identify if there were any citations that included, at least, two of our keywords. The second step consisted of reading the abstract to evaluate if the publication was related to the effect of fungi on ants, and to separate experimental papers from reviews or book chapters (the last two were not selected due to not having experimental data and/or not having the details needed for experimental data analyses). Up to this point, we looked at 2,677 citations. The third step entailed looking for the complete publication of the selected experimental studies, downloading, and reading them, to further select the ones dealing with the use of fungi in the biological control of pest ants. The fourth step involved looking for publications related to the biological control of ants by fungi cited as references in the selected papers (from step 3), as well as those cited in reviews (step 2), downloading and reading them, and including them if appropriate in our group of selected publications. This step allowed us to include relevant data published in years previous to 2000 (10 publications out of 28 read). If the study (steps 3 and 4) was written in other languages than Spanish, English, Portuguese, or French, we used Google Translate to understand it and decide about its inclusion in the list of selected publications. Overall, we read 265 publications from end to end. The fifth step required removing the studies that did not meet the minimum criteria for their consideration, such as not having a control group or studies that showed data obtained via unorthodox ways (for example, by farmers). Nevertheless, we kept some publications that did not have statistical analyses because we considered them relevant, either for being a rarely studied species or being a field study. Therefore, here, we included data from 85 publications in total (**Figure 1**), from which 65 are published papers, 18 are theses, and two are proceedings. We have not included publications that combined an entomopathogen with another type of non-biological control.

It is important to highlight that some publications have included several fungal strains, and/or ant species, or have used more than one methodology in their experimental designs, and/or presented data from more than one experiment; therefore, that publication was counted more than once, and the sample sizes varied accordingly. We defined an experiment when a study with an inoculated group with one or more treatments, was done simultaneously with a control group. However, when we quantified the number of times each fungal genus was used, we counted each treatment from every experiment as one, in order to have an idea of how often that fungus was tested. In addition, some experiments did not have all the information we used in our comparisons. These situations produced different sample sizes selected from the entire data set that complied with our criteria (step 5).

After finishing step 5, we listed the ant pest species found, and for each of them, we looked for their common names (besides their Latin binomial), their status as pest and/or invasive, the place of origin of the species, and their actual distribution (**Table 2**). In addition, we counted the number of times a fungal genus, or an ant group (LCA, *Solenopsis*, and Others), was found in each selected publication, taking into account the type of experiment (laboratory or field).

**Table 2:** Status, native places, and actual distribution of ant species considered pest and/or invasive from which fungal biological control has been studied.

Ant group/Common name	Ant species	Status	Native places	Actual distributions
Leaf cutter ant (LCA)	*Acromyrmex ambiguus*	pest	Southern South America	Southern South America
	*Acromyrmex crassispinus*	pest	Southern South America	Southern South America
	*Acromyrmex heyeri*	pest	South America	South America
	*Acromyrmex landolti fracticornis*	pest	South America	South America
	*Acromyrmex lobicornis*	pest	South of South America	South of South America
	*Acromyrmex lundii*	pest	South of South America	South of South America
	*Acromyrmex pubescens*	pest	South of South America	South of South America
	*Acromyrmex subterraneus*	pest	South America	South America
	*Amoimyrmex striatus (=Acromyrmex striatus)*	pest	South of South America	South of South America
	*Atta bisphaerica*	pest	Brazil	Brazil
	*Atta cephalotes*	pest	North of South America, Central America	North of South America, Central America
	*Atta colombica*	pest	North of South America, Central America	North of South America, Central America
	*Atta laevigata*	pest	North of South America	North of South America
	*Atta mexicana*	pest	Central America	Central America
	*Atta sexdens (=Atta sexdens piriventris, =Atta sexdens rubropilosa; =Atta sexdens sexdens)*	pest	South America	South America
Fire ant	*Solenopsis invicta*	pest and invasive	Central of South America	North America, Central America, South America
	*Solenopsis geminata*	pest and invasive	Central America	North America, Central America, South America, Oceania, Southeast Asia, South Asia, Central Africa, West Africa, Greece, Italy,
	*Solenopsis saevissima*	pest and invasive	Central of South America	South America, Africa
Carpenter ant	*Camponotus pennsylvanicus*	pest and invasive	North America	North America, Bermuda
Black garden ant	*Lasius niger*	pest and invasive	Europe and East Asia	Europe, North Asia, Central Asia, East Asia, North America
Flower ant	*Monomorium floricola*	invasive	Southeast Asia	North America, Central America, South America, South Asia, Southeast Asia, Oceania, Southern Africa, East Africa, Central Africa, United Kingdom
Common red ant	*Myrmica rubra*	invasive	Europe and North Asia	Europe, North Asia, Central Asia, East Asia, North America
Long-legged ant	*Anoplolepis gracilipes*	pest and invasive	Southeast Asia	Oceania, Southeast Asia, South Asia, Central America, Chile, United Kingdom, South Africa,
Black ant	*Dolichoderus thoracicus*	invasive	Southeast Asia	Southeast Asia, Africa, Europe, Hawaii

### 2.2 Publications from laboratory experiments

We calculated the number of times a fungal genus/species or an ant group/genus (LCA, *Solenopsis*, and Others) was found in each selected publication, taking into account the origins of the fungal strain used (unknown, soils, collections, commercial products, or different or same host as the target ant), the concentration of fungus used, and the technique for its inoculation (topical application of fungal suspension over each ant, ants walking on surface with conidia, spraying a solution with conidia over the ants or colonies/subcolonies, immersion of ants in a fungal conidia suspension).

We calculated the percentage of ant net mortality (NM) by subtracting the value of the control group from the inoculated one. Similarly, we calculated the percentage of confirmed ant net mortality (NCM) considering the percentage of ant cadavers from which the inoculated fungi were recovered as the cause of death.

From the laboratory data set we made a subset based on two conditions: on one hand, the same experiment should have reported data for NM and NCM, and on the other, it should have been replicated at least twice. Because different records reported information for some of the independent variables (e.g., strain origin is not reported but the inoculation technique it is) the sample size of the data subset varied depending on each of the variable considered. When statistical analyses were possible, we used non-parametric tests. We used Kruskall-Wallis for mortality comparisons among more than two groups, and Mann-Whitney for contrasts. The value of alpha (0.05) was adjusted using the Bonferroni correction (Siegel and Castellan, 1974).

The laboratory data set included the following references (see **Supplementary Materials Table S1**): Alves et al. (1988); Sanchez-Peña (1992); Siebeneicher et al. (1992); Pereira et al. (1993); Stimac et al. (1993a); Stimac et al. (1993b); Kelley-Tunis et al. (1995); Ortiz and Orduz (2001); Lopez and Orduz (2003); Reynolds and Currie (2004); Loureiro and Monteiro (2005); Silva et al. (2006); Sulistyowai et al. (2006); Varón Devia (2006); Santos et al. (2007); Castilho et al. (2010); Evans et al. (2010); Mena Córdobaa (2010); Cadavid-Flórez (2011); de Oliveira et al.(2011); Folgarait et al. (2011b); Folgarait et al. (2011a); Folgarait et al. (2011b); Goffré (2011); Hu et al. (2011); Kafle et al. (2011); Fan et al. (2012); Folgarait et al. (2012); Jin et al. (2012); Nagamoto (2012); Ribeiro et al. (2012); Zarzuela et al. (2012); Qiu et al. (2014); Tiscornia et al. (2014); Wallace et al. (2014); Caldwell (2015); Cooling (2015); Goffré and Folgarait (2015); Mesén-Porras (2015); Castrillo et al. (2016); Dornelas et al. (2016); Li et al. (2016); Marfetán (2016); Barcoto et al. (2017); Canali (2017); do Nascimento et al. (2017); Dornelas et al. (2017); Polezel (2017); Rocha et al. (2017); Varanda-Haifig et al. (2017); Bezerra (2018); Bizarria et al. (2018); Goffré et al. (2018); Rojas et al. (2018); Fernandez-Daza et al. (2019); Folgarait et al. (2019); Jaccoud et al. (1999); Kermarrec et al. (2019); Qiu et al. (2019); Silva (2019); da Silva et al. (2020); Folgarait et al. (2020); Santos et al. (2020); Stefanelli et al. (2020a); Stefanelli et al. (2020b); Valencia-Giraldo et al. (2020); Folgarait and Goffré (2021a); Folgarait and Goffré (2021b); Lin et al. (2021); Mendonça et al. (2021); Mendonça et al. (2021); Mota Filho et al. (2021); Cardoso et al. (2022); Park et al. (2022).

### 2.3 Field studies publications

As for field studies, our criteria for selecting a publication included, at least, to have a control group simultaneously evaluated with the inoculation treatment/s, a sample size greater than one, and results gathered by scientists. This criterion included several studies of different quality, but we purposefully included them in order to discuss them and give future directions about the control of pest ants. From these, we calculated and compared the percentage of colonies controlled by subtracting the mortality of the control group from the inoculated one from each experiment found that had not considered the inactivation of the control group. We also summarized the inoculation techniques used and their frequency of applications and amounts applied, fungal species/strain used, the concentration of conidia, the source of the strain, and the criteria of ant or mound inactivation with their respective results. No statistical analyses were done due to the few studies published and the differences in experimental designs and data collected.

The field studies publications included in this section were: Stimac et al. (1989); Diehl-Fleig et al. (1993); Oi et al. (1994); Álvarez and González (2002); Bextine and Thorvilson (2002); Lopez and Orduz (2003); Banderas (2004); Barrera Llano (2006); Varón Devia (2006); Amarilla Salinas and Arias Ruiz Díaz (2011); Kafle et al. (2011); Tiscornia et al. (2014);Hernández (2016); Li et al. (2016); Rojas et al. (2018); Dávila Pino (2018); Fernandez-Daza et al. (2019); Folgarait (2019); Nalini and Sasinathan (2020); Folgarait and Goffré (2021a) (see **Supplementary Table S2**).

## 3 Results

### 3.1 Publications on ant control by fungi

**Figure 2:**
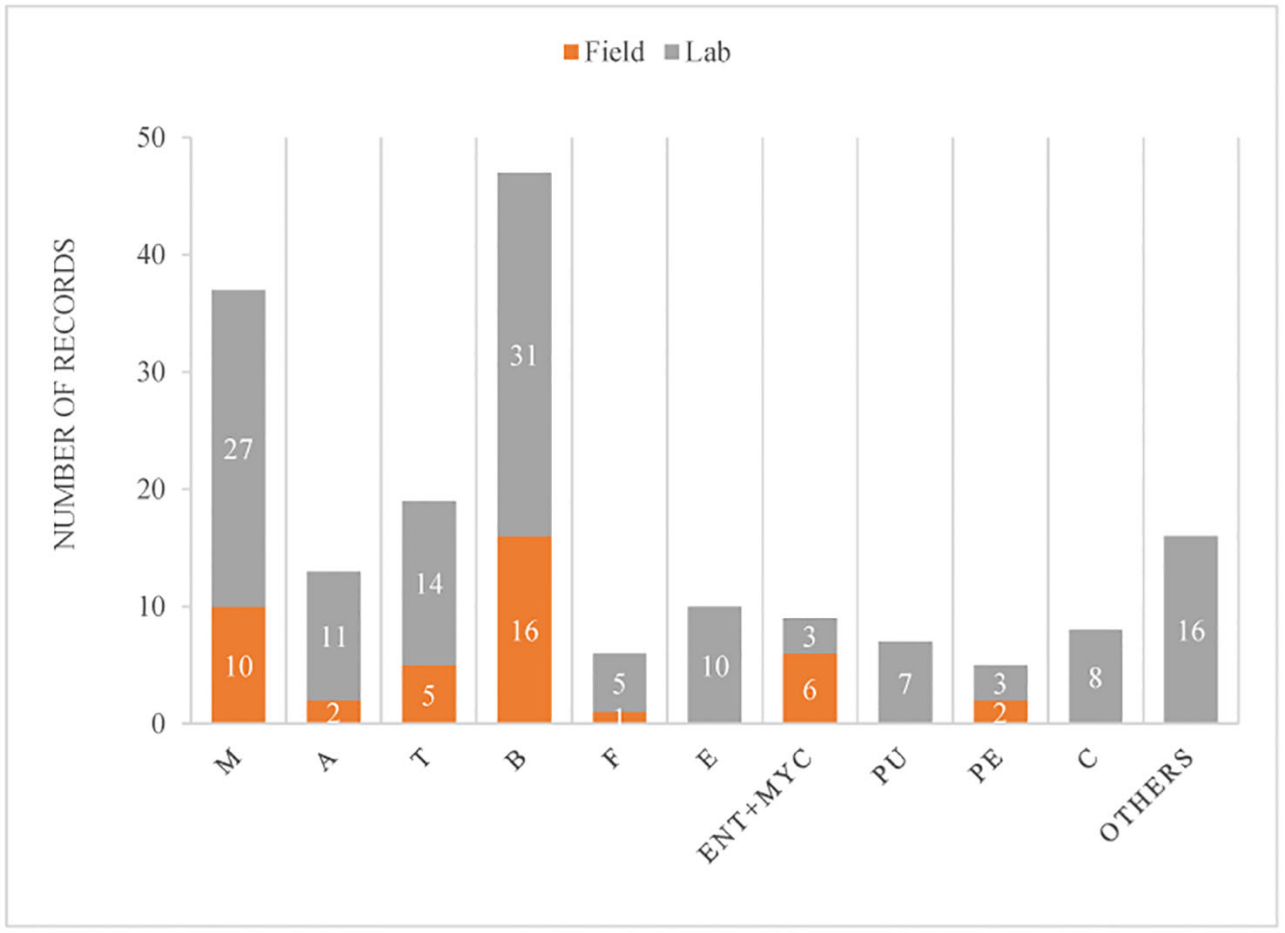
Number of records in which fungal genera were tested against ants or their cultivated fungi (in the case of LCA), discriminated according to being evaluated in the laboratory or field. Fungal genera considered: M, *Metarhiziu*m; A, *Aspergillu*s; T, *Trichoderma*; B, *Beauveria*; F, *Fusarium*; E, *Escovopsis*; ENT+MYC, the combination of an entomopathogen (*Beauveria* or *Metarhizium*) with a mycoparasite (*Trichoderma*); P, *Purpureocillium*; Pe, *Penicillium*; C, *Cordyceps*; and Others, included 12 others fungal genera.

A total of 85 publications were included in this study. After their discrimination by fungal genus, and whether they were tested in the laboratory or field, we ended up with 177 records from those publications, where *Beauveria* was the fungal genus most tested (27%), followed by *Metarhizium* (21%) (**Figure 2**). The great majority were done in the laboratory (77%).

From all the publications selected, 82 out of 85 mentioned the origin of the strain used for inoculation. Considering that a same publication could have more than one strain with different origins, from the 82 publications 38% utilized strains isolated from the same ant species as the target to be controlled (called from now on as the target ant species or same ant host), 30% corresponded to strains obtained from another ant host (from now on reported as different ant host species), 20% used commercial products, 16% used a strain which was collected from a non-ant insect host, 15% were obtained from fungi collections, and 12% used strains collected from soil samples.

Regarding the concentration, 72 publications informed the concentration used for the fungal strains tested; in the rest, it was expressed in such a way that it could not be estimated. The concentration 1 × 10^8^ conidia/ml (or g) and tests with more than one concentration for a same fungal strain, were the most used, each representing 24% of all publications. Concentrations in the order of 10^7^ conidia/ml (or g) followed with 18%, and in the order of 10^6^ with 15%. The orders of 10^3^, 10^4^, 10^5^, 10^9^, 10^11^ and 10^12^ conidia/ml or conidia/g were shown each in less than 7% of the papers.

The inoculation techniques applied varied depending on whether the study was carried out in the laboratory or in the field, therefore were detailed in the following sections.

Most of these studies (67%) corresponded to LCA, whereas 24% were about fire ants, and 9% included other ant pest species.

### 3.2 Laboratory studies

**Figure 3:**
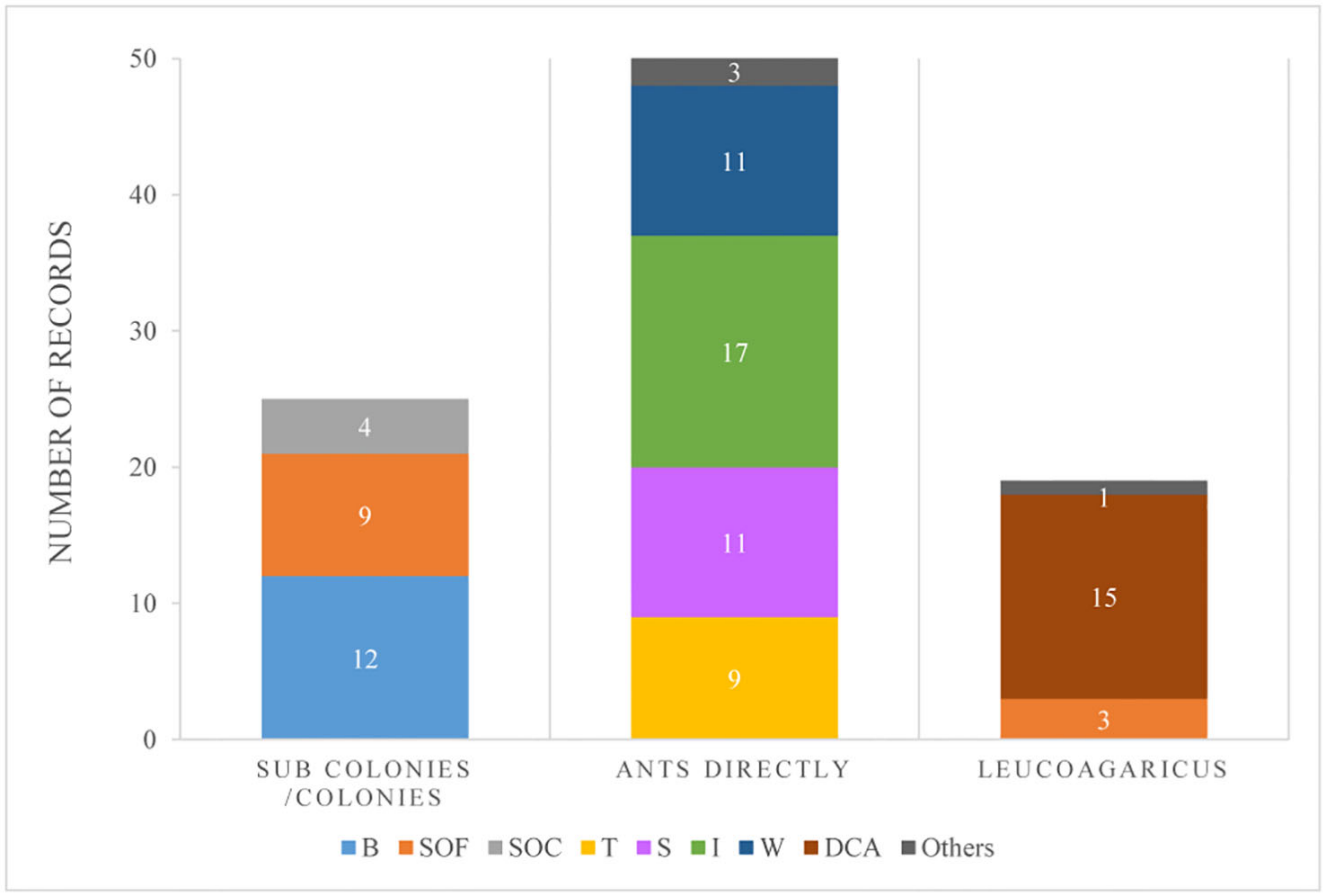
Number of records from laboratory experiments for different methods of fungal inoculation discriminated by its application on sub-colonies or entire colonies, or applied directly on the ants or the cultivated fungi (*Leucoagaricus* sp., in the case of LCA). Types of methods considered: B: baits; SOF: fungal conidia spray over the cultivated fungi (in the case of LCA); SOC: fungal conidia spray over the ants from a colony/subcolony; T: topical application of fungal suspensions on each ant; S: fungal suspensions sprayed over ants; I: immersion of ants on a fungal suspension; W: ants walking on a surface previously covered with conidia; DCA: dual culture assays against *Leucoagaricus* sp.; Others: such as feeding, conidial shower, etc.

The selected laboratory publications included 324 strains, which corresponded each to different strains. In addition, we registered 3 records of a combination of two different strains used simultaneously (see **Supplementary Table S1**). Those strains were obtained from 72 publications performed in the laboratory (**Supplementary Table S1**) in which 95 records of techniques used for inoculation were reported. Most of these (54%) evaluated ant mortality by inoculating ants directly, and within this group, the techniques of ant immersion or spray were the most commonly used (39% and 27% respectively). Only 26% of all records had inoculated the ants within a colony or sub-colony (**Figure 3**).

From a total of 60 papers that provided information on fungal concentrations (conidia/g or conidia/ml) used for inoculation, and that were discriminated by ant groups, we found that most papers (27%) corresponded to testing several concentrations for a same strain, followed by 22% that used a concentration in the order of 10^8^ conidia/ml or g, 17% used inoculums in the order of 10^6^, 14% in the order of 10^7^ whereas a 20% included several other concentrations hardly replicated across papers. Consistent with previous results, most of these records have LCA as the target (54%), while *Solenopsis* sp. represented 34%, and other ant species 12%. When the concentration was discriminated by fungal species, we obtained 115 records from which the great majority corresponded to *Beauveria* (32%), followed by *Metarhizium* (22%) strains, and the rest of the fungal genera represented each less than 10% (**Supplementary Table S1**).

We found 151 records that reported the origin of the fungal species. In 29% of them, the strain used belonged to the same target ant species, a 24% corresponded to strains isolated from different ant host species, 16% were obtained from another non-ant insect species, 11% represented strains collected from the soil or plants, an additional 11% were gathered from fungal collections, and a 6% from commercial products; only for 3% of the records the origin of the strain was not reported.

**Figure 4:**
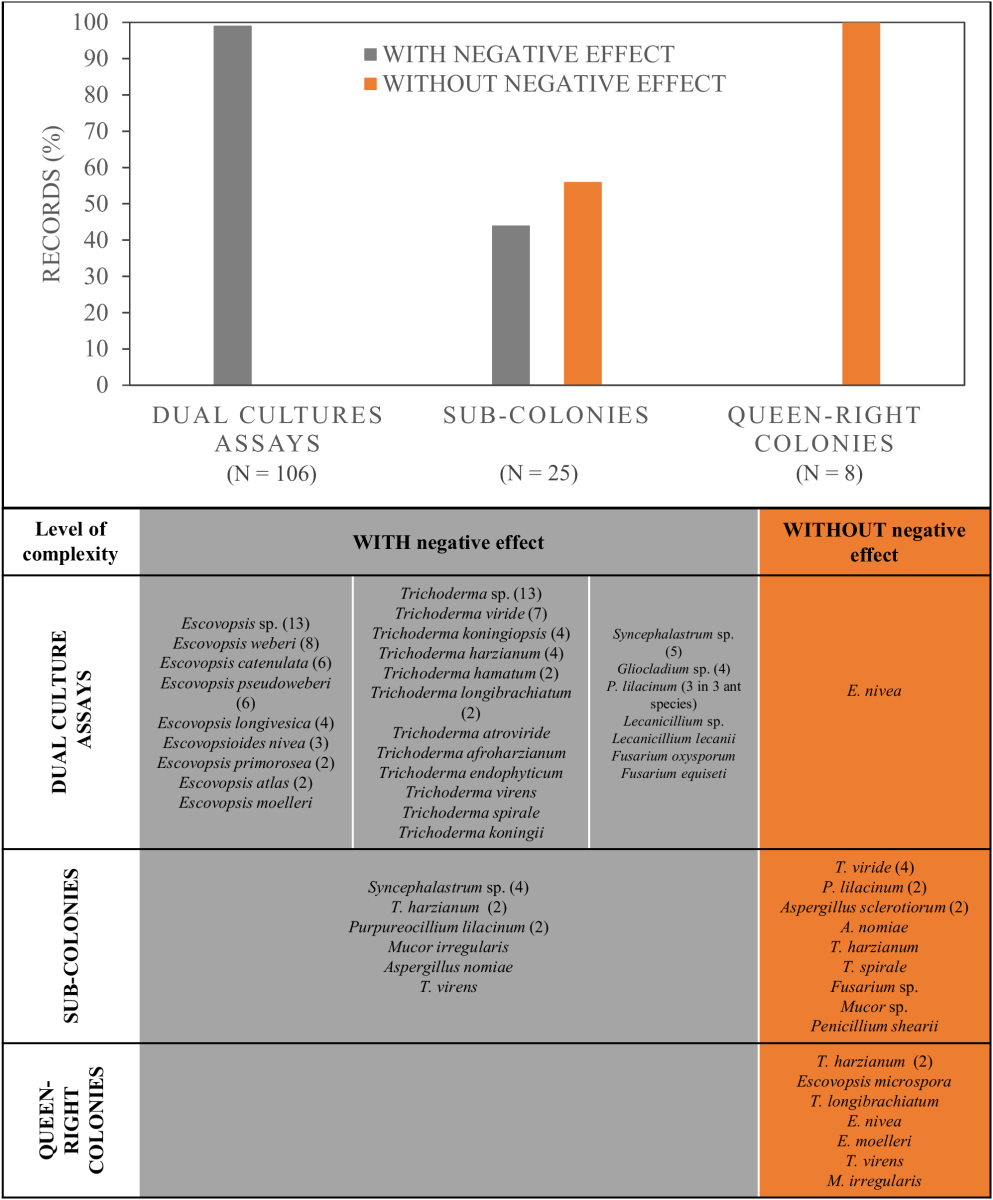
Percentage of records that produced a negative effect or not over the growth and health of *Leucoagaricus* sp. in experiments with different colony components, from simpler to more complex: *Leucoagaricus* grown without ants in dual culture assays, laboratory sub-colonies with fungus garden and workers, and laboratory colonies, with fungus garden, queen and workers. Below, the identity and number of fungal strains used for each type of experiment.

For LCA, many publications considered the use of a mycoparasite, or fungal antagonist, to evaluate its effect on the fungal cultivar (*Leucoagaricus* sp.) that LCA used as their food. Within this latter group, which included 19 publications encompassing 18 fungal species strains and morphotype strains, 76% were done *in vitro*, using dual cultures whereas the rest used sub-colonies or queen-right colonies (**Figure 3**). Different mycoparasites were used with the latter method, from which 45% corresponded to *Escovopsis* spp. (8 different species and 13 morphotypes, totalizing 45 strains), 38% to the genus *Trichoderma* (11 different species and 13 morphotypes, totalizing 38 strains), whereas 16% used other fungal genera (**Figure 4**). In 99% of all the strains tested in dual cultures, a negative effect over *Leucoagaricus* was registered (**Figure 4**). However, for only 35 strains (33%), the antagonism was studied under the microscope in order to confirm if there was cell wall degradation of the ant mutualist fungi, or if specialized parasitic structures were developed by the antagonistic fungi, etc. (**Supplementary Table S1**).

Regarding the effect of mycopathogens over *Leucoagaricus* tested in sub-colonies, we found 25 records (see table from **Figure 4**). The latter represents laboratory colonies artificially assembled which had workers and their mutualistic fungi. All, except 3 studies that used baits, applied a suspension of conidia directly over the *Leucoagaricus*. The mutualist fungi came from three ant species, where *Atta sexdens* was the main species used (**Supplementary Table S1**). From these studies, a 44% registered a negative effect. This effect was produced by 12 strains belonging to mycopathogens from five species and one morphotype (**Figure 4**). A negative effect was only found over *Leucoagaricus* from *At. sexdens*. In these cases, the most common origin of the mycopathogen was the same ant host. A slightly greater percentage (56%) of records did not find a negative effect of the mycopathogen suspension on the *Leucoagaricus* fungus. This set included seven fungal species and two morphotypes, encompassing 13 strains (see table from **Figure 4**). It is important to highlight that for three species that were tested more than once (*Purpureocillium lilacinum*, *Trichoderma harzianum*, and *Aspergillus nomiae*), different effects were found, meaning that different strains from a same fungal species showed a negative effect or no effect.

We found eight records that used laboratory colonies, which included the queen. In none of them there was a negative effect on the ant colony despite the direct application of the antagonistic fungal suspension over the *Leucoagaricus* (**Figure 4**). Seven species were tested, including three *Escovopsis*, three *Trichoderma* and one *Mucor* species (see table from **Figure 4**). Five of them were tested on *At. sexdens* and three on *Acromyrmex subterraneus*. In these cases, the most frequent origin of the mycopathogen was the same ant host (**Supplementary Table S1**).

#### 3.2.1 Comparisons of ant mortality: all strains

From the initial 362 records, including either different or same strains used more than once, we selected those that were entomopathogenic (222 records). From these we further selected 125 which represented those experiments that reported data on net mortality (NM) as well as on net confirmed mortality (NCM). Depending on the variable analyzed, the data set consisted of 121 to 125 records. Among those records, 49% tested *B. bassiana*, 46% were tested on *At. sexdens*, 64% of the strains were isolated from the target ant species, 78% used the immersion technique for inoculation, and 58% used a concentration of 1 × 10^8^ conidia/ml (**Supplementary Table S1**).

**Figure 5:**
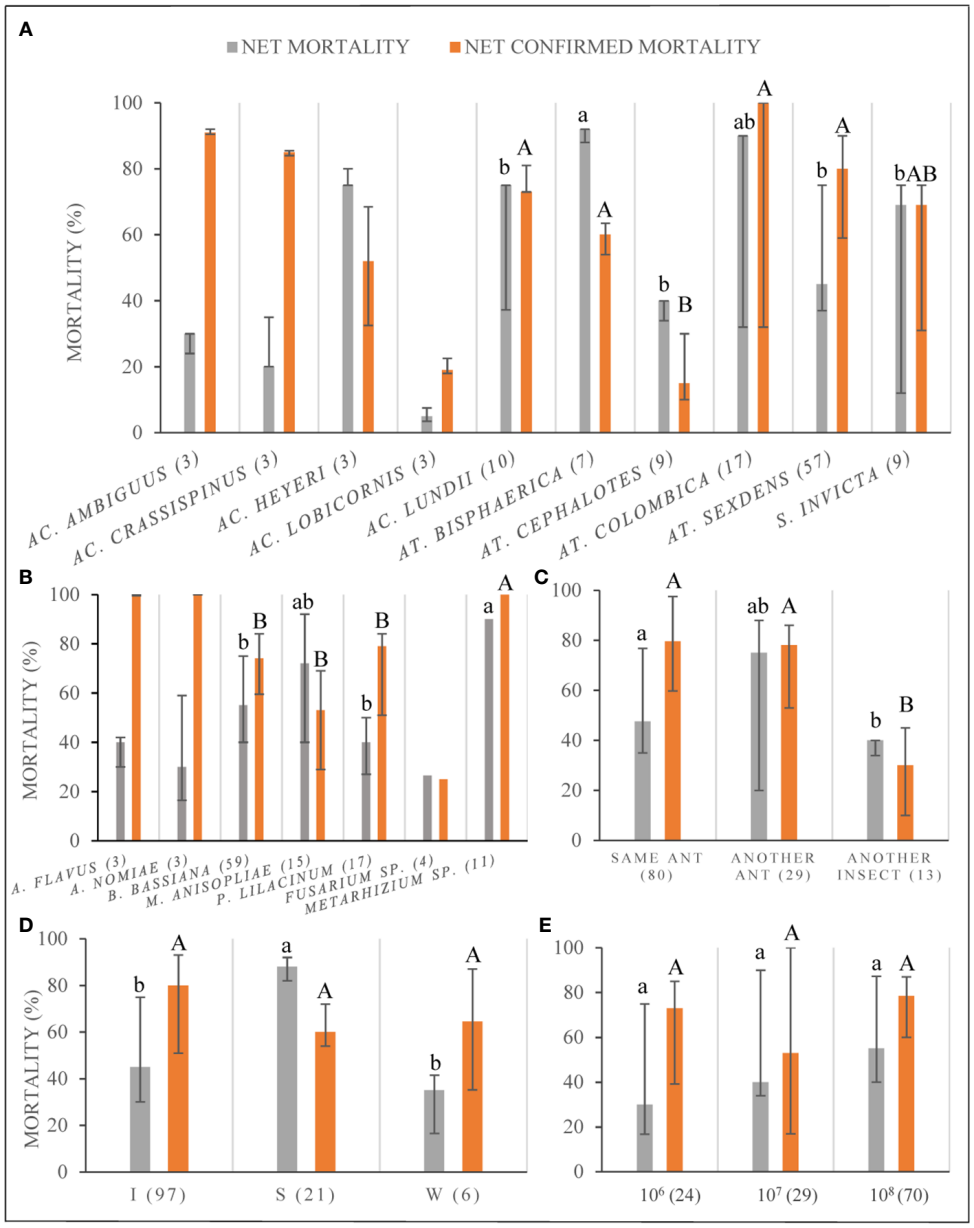
Percentage of ant net mortality (NM) and net confirmed mortality (NCM) discriminated by: **(A)** different ant species; **(B)** different fungal species; **(C)** fungal strain origin; **(D)** type of fungal inoculation, as I: immersion of ants on a fungal suspension S: fungal suspensions sprayed over ants; W: ants walking on a surface previously covered with conidia; and **(E)** fungal concentration of the inoculum. Data shown as medians with quartiles. The sample size for each variable is shown in parentheses. Lowercase letters for statistical comparisons among NM, and capital letters among NCM. Different letters imply significant differences among treatments, for the corresponding significance value according to the Bonferroni correction.

The NM varied significantly among ant species (KW=19.6, df=5, *p < 0.001*; only considering those species with N>5) being around 90% for *Atta bisphaerica* and *Atta colombica* and between 70-75% for *Acromyrmex heyeri*, *Acromyrmex lundii*, and *S. invicta*; the first two ant species had significantly (*p < 0.01*) greater values of NM than the others (**Figure 5A**). For the mentioned species, the NCM was either high or intermediate (**Figure 5A**), and also differed significantly among ant species (KW=19.6, df=5, *p < 0.001*). When the analyzed records only included *M. anisopliae* or *B. bassiana*, the greatest values of NM were registered for *At. bisphaerica* and *At. sexdens* for the first fungi whereas for the second one, the greatest percentage of NM was found in *At. bisphaerica*, *Ac. heyeri*, *Ac. lundii*, and *S. invicta*.

The NM varied significantly among fungal species (H=26.1, df=3, *p < 0.0001*; only considering those species with N>5) being significantly higher for *Metarhizium* sp. than *P. lilacinum* and *B. bassiana* (both contrasts *p < 0.0125*), but not different compared to *M. anisopliae* (*p = 0.83*) (**Figure 5B**). The greatest values of NM corresponded to *Metarhizium* sp. with a median of 90%, followed by *M. anisopliae* with a median of 70%. The NCM also varied significantly among fungal species (H=29.8, df=3, *p < 0.0001*; only considering those species with N>5) and *Metarhizium* sp. had significantly higher NCM than other species (*p < 0.0125*), with a median of 100% (**Figure 5B**). All data on *Metarhizium* sp. belong to different strains tested under the same conditions published in one publication.

Regarding the mortality by strain origin, the greatest values were found when the strain origin was from another different ant host species and the lowest when the strain came from a non-ant species (**Figure 5C**). However, the comparison was not significantly different although borderline (H=20.8, df=2, *p = 0.054*). The same pattern was found when looking only at the *B. bassiana* data set; however, when data only from *M. anisopliae* was analyzed, great values of NM were also observed when the origin of the strain was the same ant species as the target. Nevertheless, the variation was much greater when the strain was obtained from different ant host species (**Supplementary Table S1**). There were significant differences of NCM across strains with different origins (H=20.8, df=2, *p < 0.0003*); these were explained by the significantly lower values of NCM when the strain used came from another non-ant insect (both contrasts *p < 0.0002*) (**Figure 4C**).

The ant mortality varied significantly (KW=31.2, df=2, *p < 0.00001*) among inoculation techniques, being significantly greater when the ants were sprayed with the fungal suspension than with any other technique (*p < 0.0009* for both contrasts, see **Figure 5D**); however, the percentage of NCM did not differ statistically among techniques (H= 4.2, df=2, *p > 0.05*). This same pattern was observed when only *B. bassiana* or *M. anisopliae* data was analyzed (**Supplementary Table S1**).

When the concentration of the fungal suspension was considered, we did not find any significant difference among the three concentrations most frequently used (**Figure 5E**), neither for NM (H=5.9, df=2, *p > 0.053*) nor for NCM (H=3.1, df=2, *p > 0.21*). The same pattern was found for data that considered only *B. bassiana*. In the case of *M. anisopliae* only two concentrations were used and the greater gave greater mortality (**Supplementary Table S1**). However, when we analyzed data obtained from papers that had values of mortality under different concentrations for the same strains, we did find a pattern of greater NM when the concentration increased (y = 6.1941x + 31.343; R² = 0.9327) (those papers included concentrations from 1 × 10^4^ to 1 × 10^8^ conidia/ml; there was information for three other concentrations but these were not considered because there was only one data for each concentration) (**Supplementary Table S1**).

#### 3.2.2 Comparisons of ant mortality: only strains used more than once

The comparison of NM using the same strain (under the same conditions) in different ant species (N = 19) showed that for ten strains the mortality was very similar between related ant species, for five strains the mortality was equal or greater than 10% but lower than 35%, and for four strains the percentage of NM changed even more across different hosts, between 40 to 70%. The latter corresponded to *Acromyrmex* and *Solenopsis* species. However, in one case, the same strain (Bb447) used with *S. invicta* changed a lot when it was tested on a LCA species (from a median of 85% of NM for S. invicta to 5% for LCA), but when it was tested on *Solenopsis saevissima* the change was small (82% of NM) (**Supplementary Table S1**).

Another analysis was made when the NM produced by the same eight strains was compared before and after being re-isolated from *At. sexdens* (all comparisons under the same conditions). A change smaller than 10% in mortality was registered for two strains whereas a change of 10% was registered for 13 strains, and a change of 17% was registered for the strain *B. bassiana* BBOT04, and of 28% for *P. lilacinum* PBOT38. The 16 comparisons corresponded to a single paper (Cardoso et al., 2022).

### 3.3 Field studies

**Figure 6:**
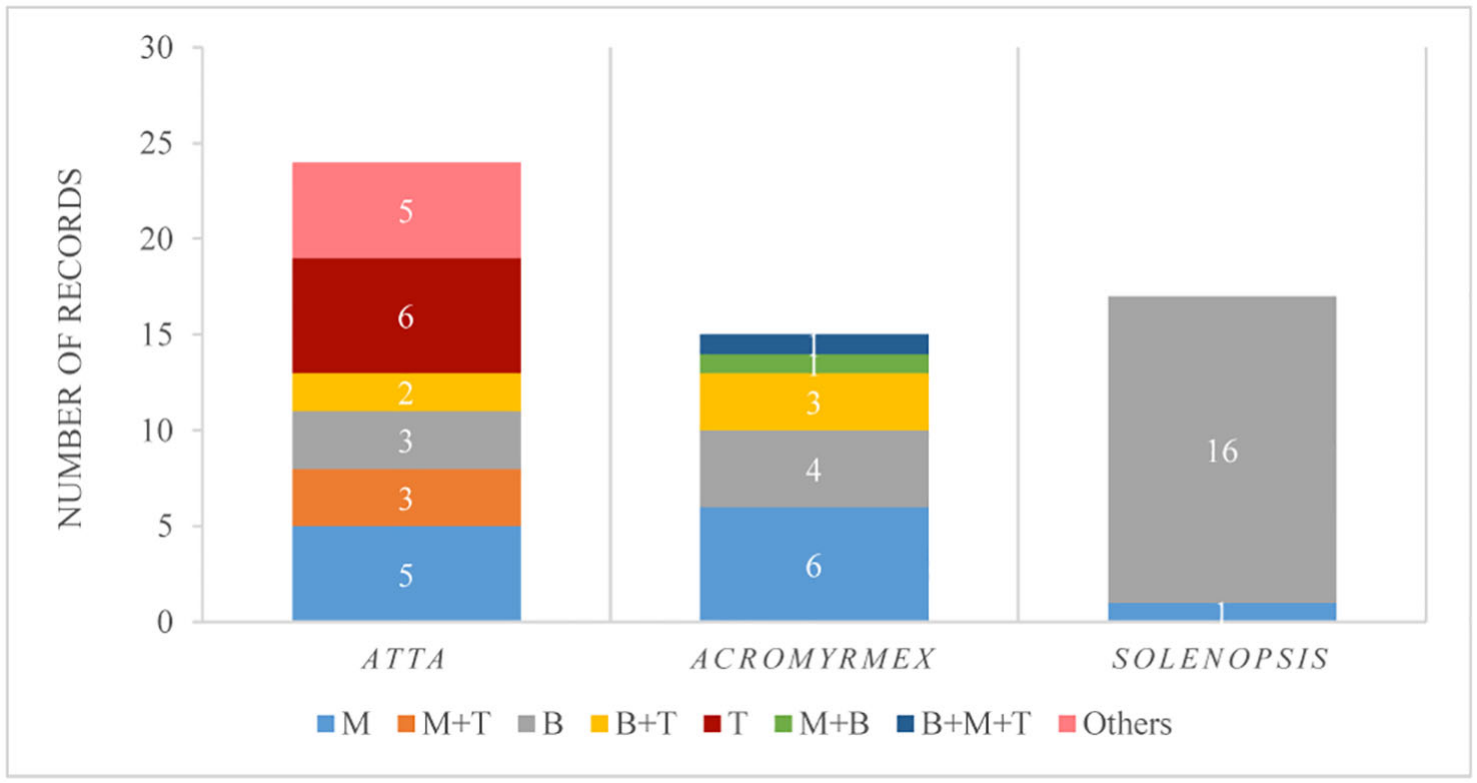
Number of records in which a fungal genus was tested in the field, discriminated by ant genus. Fungal genera considered: M, *Metarhizium anisopliae*; M+T, *M. anisopliae* and *Trichoderma* sp.; B, *Beauveria bassiana*; B+T, *B. bassiana* and *Trichoderma* sp.; T, *Trichoderma* sp.; M+B, *M. anisopliae* and *B. bassiana*; B+M+T, *B. bassiana*, *M. anisopliae* and *Trichoderma* sp.; Others, *Purpureocillium* sp.*, Purpureocillium* sp.*+Trichoderma* sp.*, Aspergillus flavus, Penicillium* sp*, A. flavus+Penicillium* sp.

Twenty publications meet our criteria, 12 were done with LCA (six for *Acromyrmex* and six for *Atta* species), seven with *Solenopsis* (five with red fire ants, *S. invicta*, one with *S. saevissima*, and one with *S. geminata*), and one with *Lasius niger*. Overall 14 pest species were evaluated, three *Atta* species, seven *Acromyrmex*, three *Solenopsis*, and one *Lasius* (**Supplementary Table S2**). Considering that some of the publications had more than one experiment, 33 experiments in total were considered. Furthermore, each of the publications usually tested more than one fungal species, evaluated either as different treatments or under different experiments. Therefore, from 56 records, *B. bassiana* was the species most tested (41%), followed by *M. anisopliae* (21%), then by *Trichoderma* spp. (11%), whereas *B. bassiana* or *M. anisopliae* in combination with *Trichoderma* sp. were found in 9 to 5% of those records, respectively, and the rest of the fungi, or their combination, were represented by less than 2% each (**Figure 6**). The number of records for *Atta* was greater than for *Acromyrmex* and *Solenopsis*. Considering only *Atta*, the combination of two fungi, and the use of *M. anisopliae* or *Trichoderma* spp. singly were evaluated more (20 to 25%) than *B. bassiana* (12.5%). Considering only *Acromyrmex*, the fungus mostly studied (40%) was *M. anisopliae*, and considering only *Solenopsis*, 94% of the records corresponded to *B. bassiana* (**Figure 6**).

Depending on the number of variables reported, there were between 30 to 33 experiments. Regarding the method of fungal inoculation in *Atta*, *Acromyrmex* and *Solenopsis*, the use of powder was the most common (39%), followed by baits (34%) but mainly because both being predominant in experiments with *Solenopsis* (54%), whereas for the other two groups of ants (*Atta* and *Acromyrmex*), all the techniques used (bait, liquid, powder) were similarly represented. For *Atta*, the number of experiments with a length of four to seven weeks was the same than those that lasted from eight to 14 weeks, whereas for *Acromyrmex*, the shorter ones were most common (54%), and, for *Solenopsis*, experiments with intermediate length were more represented (66%). For only four experiments with LCA species, the total duration was greater than 20 weeks. Studies using fungal strains obtained, or re-isolated, from the same host as the target ant species, were the most used (33%). Strains from different sources were used in all ant groups, but the most common origin (48%) corresponded to strains coming from an ant species, equal or different than the target one, especially so for *Solenopsis*. The use of strains from commercial products followed, shown in 15% of the experiments, partitioned equally among the three genera. Strains with a soil origin were only used with *Acromyrmex* species representing a 15% from all the experiments. For *Atta*, the strain origin was similarly (14-28%) represented among different categories. *Atta* species were inoculated using concentrations around 1 ×10^9^ conidia/g/ml in 50% of the experiments; for *Acromyrmex*, a concentration in the order of 10^10^ conidia/g/ml was used in 40% of the experiments, whereas for *Solenopsis*, most studies (73%) did not report that information (**Supplementary Table S2**). Among the 33 experiments, 11 used different amounts of product depending on the nest size. In addition, considering those experiments that reported mound inactivation, most applied the biological product once (N= 19) or three times (N= 15) with means (and standard deviations) of 43,8% (33.4) and 66% (28.5) of mound inactivation, respectively (**Supplementary Table S2**).

**Figure 7:**
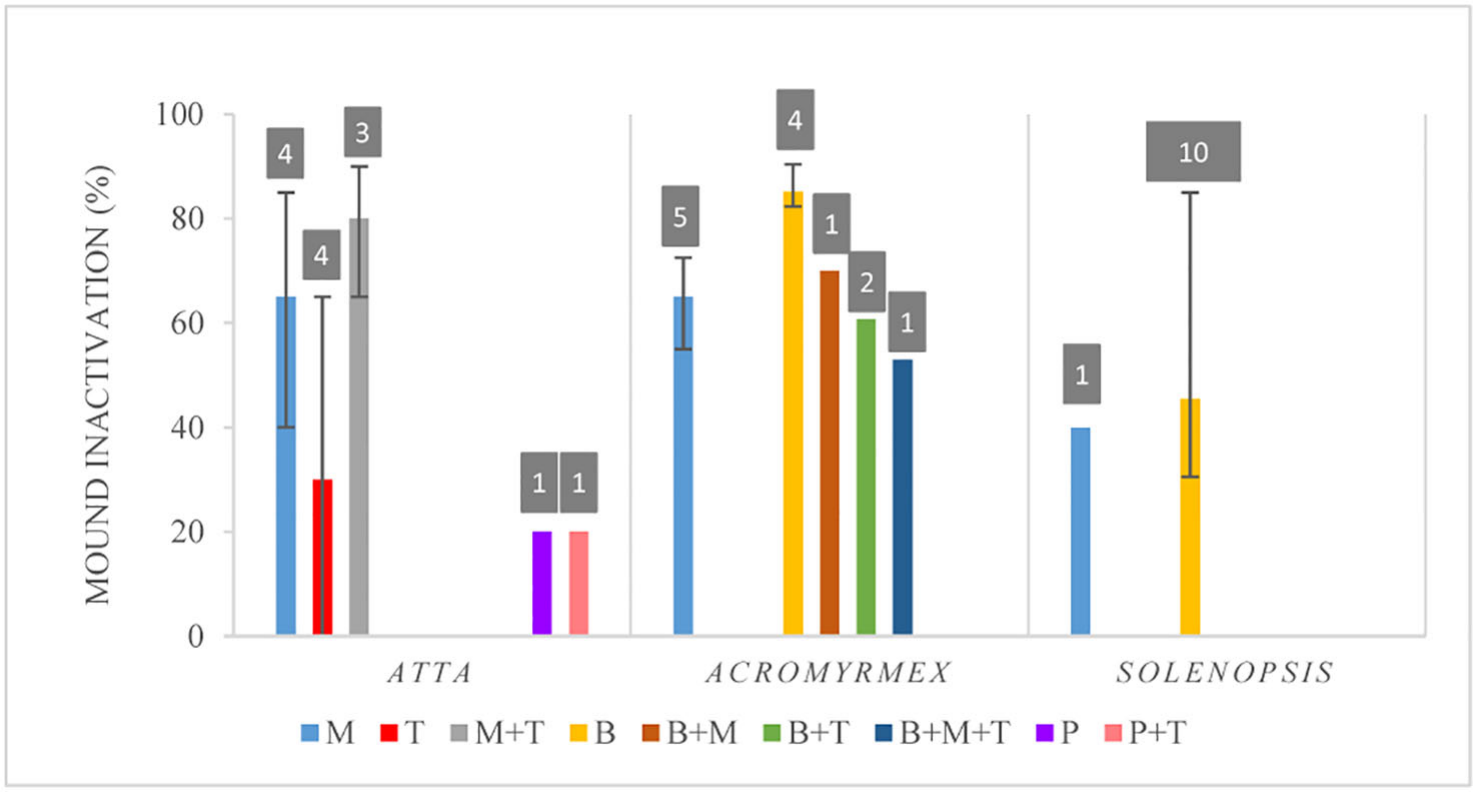
Percentages of mound inactivation discriminated by fungal genera. Data shown as medians with quartiles. Fungal genera considered: M, *Metarhizium anisopliae*; T, *Trichoderma* sp. M+T, *M. anisopliae* and *Trichoderma* sp.; B, *Beauveria bassiana*; B+M, *B. bassiana* and *M. anisopliae*; B+T, *B. bassiana* and *Trichoderma* sp.; B+M+T:,*B. bassiana*, *M. anisopliae* and *Trichoderma* sp.; P, *Purpureocillum*; P+T, *Purpureocillum* sp. and *Trichoderma* sp. Numbers above the bars, in grey squares, show the number of experiments that included each fungal treatment.

Finally, for *Acromyrmex*, all studies reported mound inactivation with values ranging from 20% to 100%. In the case of *Solenopsis*, 97% of the studies reported mound inactivity, which went from 6% to 100%. For *Atta*, 43% of the experiments showed values of mound inactivation going from 0% to 100%, whereas the rest reported a reduction in ant flow for the inoculated nests in comparison to the control groups (**Supplementary Table S2**). **Figure 7** shows the median values, and quartiles (1 and 3) of the percentages of mound (colony) inactivation discriminated by fungal genus and ant genera, which varied from 20% to 85%. Despite the use of different strains across studies, it seemed that *B. bassiana* was more effective in *Acromyrmex* whereas the combination of *M. anisopliae* with *Trichoderma* spp. was better for *Atta*. However, the number of studies that included any of the fungal treatments is quite low, except for *Solenopsis* (**Figure 7**), where two publications reported results of several slightly different experiments, all using the same strain of *B. bassiana*. Overall, the median efficiency of control calculated for all fungi together was 43% for *Atta* and 66.7% for *Acromyrmex*, whereas for *Solenopsis*, the median efficiency was 42.7% (**Supplementary Table S2**). However, the variation around the medians was quite high, especially for *Metarhizium* treatments in *Atta*.

## 4 Discussion

The biological control of pests represents an extremely important strategy nowadays, as demands from both certifying organizations and the public increase every day (Lacey et al., 2015). Pest ants do not escape from this scenario. In this review, we found that most of the work related to the control of pest ants by pathogenic fungi was performed in the laboratory and with strains of *B. bassiana*.

### 4.1 Fungal inoculations carried out in the laboratory

*B. bassiana* and *M. anisopliae* seemed to be better control agents than the other fungi that have sufficient replicates to be compared to. *B. bassiana* produced greater NM in *Acromyrmex* and *Solenopsis* (medians of 68% and 69%, respectively) in comparison to *Atta* (median of 50%), whereas *M. anisopliae* produced more NM in *Atta* (median 72%) in comparison to *B. bassiana* (no tests of *M. anisopliae* were done for the other two genera). *At. bisphaerica* seemed to be the species best controlled within *Atta*, and *Ac. lundii* within *Acromyrmex*, in addition to *S. invicta*. It seemed that it was worse to use a strain collected from a non-ant insect in comparison to strains with an ant origin. The spray technique seemed to produce greater ant mortality for inoculating the ants, and this result is promising considering that it is feasible to apply a spray in the field. Regarding concentrations, we did not find a clear pattern of higher mortality at higher concentrations for the whole data set; nevertheless, a positive relationship between both variables was found if we only considered those papers that have used the same strain with different concentrations.

Different strains, within the same fungal species or genera, had different efficiencies in terms of killing the ants. These results might not be surprising if we consider that, on one hand, we were analyzing generalist pathogens that may vary their virulence for different hosts, and on the other, there are publications acknowledging the importance of using particular strains for the control of various insect pests (Jones et al., 1996; Quesada-Moraga et al., 2006; Kafle et al., 2011; Ren et al., 2012; Valero-Jiménez et al., 2014). However, for pest ants the importance of strains has hardly been acknowledged in the literature (Goffré et al., 2018; Folgarait et al., 2020). There were few cases in which the same entomopathogenic or mycopathogenic strain was tested more than once, and we found situations where the effects were small and others that were greater, when the same strain was evaluated under different conditions or ant species. Therefore, it is difficult to anticipate for which strains one outcome, or the other, may occur. It seemed that greater changes in virulence were found when host species differed, especially so among *Acromyrmex* species in comparison to other ant genera. It will be advisable that future publications do not longer emphasize the effect of an antagonistic species and rather use the strain name because it is not clear how much is possible to extrapolate an effect at the species level. Bidochka et al. (2002) suggest that a good biological control fungal strain is that obtained from the same habitat as the target species because it will be adapted to the abiotic conditions; unfortunately, we cannot assess that hypothesis due to a lack of information from the publications.

The results on mycopathogens have shown a pattern: the lower the “complexity” in which the antagonistic fungus was tested over *Leucoagaricus*, the greater negative effect was found. In addition, we should highlight that from 67% of the records using the simplest “complexity”, i.e. dual cultures assays; it was not possible to distinguish if the negative effect was due to competition or parasitism. In turn, when all the major components of a colony - workers, mutualistic fungi and queen -, were considered, no negative effects were found. However, tests with higher “complexity” were published only for two ant species, therefore there is a need for increasing the number of studies to corroborate this pattern. If the pattern holds, it will indicate that social immunity works efficiently in defeating the antagonistic fungi when all the colony components are included, even if the mycopathogen is applied directly over the mutualistic fungi. This means that the ants would be able to remove the inoculated conidia very fast and/or weed the mycelia before harming the colony. Social immunity has been proposed for ants (Cremer et al., 2007), but seemed to be particularly effective for LCA (Currie and Stuart, 2001; Hart and Ratnieks, 2001). This pattern can also explain why the control of field LCA colonies is so difficult to achieve and requires several applications (Folgarait and Goffré, 2021a).

Several observations should be made regarding the data set analyzed, some of which could have biased the results shown. We found that NM was greater when we analyzed all the data in comparison to when we considered only those records that had replicates (i.e., assays were done using more than one colony). For example, the NM obtained with *M. anisopliae* tested on *Atta* species changed from 72% to 40% because the data set was mainly represented by experiments with one colony. Using a single colony not only poses statistical problems due to pseudoreplication, but also seems to overestimate the efficiency of the biocontrol agent. A 33% of all our records had pseudo-replicated data. Furthermore, half of them used 20 to 30 ants/treatment, which is an outstandingly low number for a test with social insects. Using higher number of colonies increases the variability of the fungal efficiency for a same target ant species (de Oliveira Augustin, 2011; Goffré and Folgarait, 2015; Folgarait et al., 2020; Folgarait and Goffré, 2021b; Cardoso et al., 2022), however, it could better demonstrate if a same strain is capable of overcoming the different resistance to infections that ant colonies have in nature. Many papers showed results from several strains tested on the same ant species and/or under the same conditions. If one of those papers represented the single source of information for a particular strain or ant species, the results could be biased (Dionisi et al., 2021). In the analyzed data set, for example, *At. colombica* had 17 records with the same NM values (90%) among strains. These records corresponded to a single publication, where most of the strains were *Metarhizium* sp., and were tested under the same conditions. Similarly, for *At. bisphaerica*, there were only 7 records, all corresponding to a same publication where the conditions under which the strains were tested were the same. Therefore, suggesting that *At. bisphaerica* is one of the best controlled ants within the genus *Atta*, could represent a questionable conclusion.

Very few studies claimed to have recovered the strain from the target host before testing its effect; this has been considered important in order to recover the virulence of the strain which could have been attenuated due to repeated *in vitro* cultures (Butt and Goettel, 2000). However, Cardoso et al. (2022), after testing 16 strains, have not found that strains which were recovered from a host were consistently more virulent than those not recovered from the target ant (the median of NM for strains before re-isolation was 55% whereas for those after re-isolation was 45%). It seems necessary to establish a protocol regarding this issue in order to have comparable data across experiments. Because different strains could be stored using several methods, could have different number of passages in culture media before its use, or used different culture media, we propose the following: always inoculate the strain in the target ant before testing it, and afterwards culture the recovered strain on PDA (which is the most commonly used media to grow fungi), and perform the necessary tests on the target ant without doing further passages. Similarly, the LD50 is another parameter that can standardize comparisons across studies with different fungal strains but this has been little done with ants (eight out of 72 publications).

Another issue was related to the report of net values of mortality; only few publications reported their data in this way. It is paramount that researchers refer to net values of mortality because if the mortality by the control group is not considered, the efficiency of the biocontrol agent could be overestimated. Lack of reporting data on confirmed mortality could give misleading results because the cause of mortality could have been something else but not the inoculated fungi. The selection of an efficient strain not only depends on its capacity to kill the ants but also on its dispersion from the ant cadaver, in order to produce horizontal transmission among the members of the ant colony. In our whole data set, only 21% of the papers (15 out of 72) reported data on confirmed mortality, and the analysis of that data showed that a high NM did not always corresponded to a high NCM (see **Figure 5**). Finally, the viability of the conidia should be assessed before its utilization; otherwise, the results could be flawed (Butt and Goettel, 2000), and this data was seldom reported in our data set.

We propose that the mentioned observations are considered in future laboratory studies not only to improve the experiments but also to standardize comparisons and reduce the variation among them.

### 4.2 Fungal inoculations carried out in the field

Most field studies have used *B. bassiana*, *Metarhizium anisopliae*, or a combination of an entomopathogen and a mycoparasite in the case of LCA, whereas *B. bassiana* was almost exclusively used for *Solenopsis* species (mainly *S. invicta*). Also, the majority of the experiments used strains obtained from the same target host or another ant, in concentrations in the order of 10^9^ or 10^10^ conidia/g or ml. Results about “ant mortality” –as mentioned in most studies– were extremely variable, but they seemed to be somehow better for *Acromyrmex*. It was clear that mound inactivation (also referred to as ant mortality) was regularly reported for *Acromyrmex* and *Solenopsis* species, whereas for *Atta*, most studies only showed a reduction in ant activity between inoculated and control groups. Both criteria could work if the results are sustained for a long period of time, but these parameters were evaluated in that way only in four publications. Although the length of those studies may seem an excessive amount of time, the main criterion to claim colony mortality is having several weeks of continuous inactivation. In our previous field study (Folgarait and Goffré, 2021b), we discussed that four weeks of continuous inactivation may be too short because the inactivation of ant colonies in the field occurs very frequently and for a variety of reasons. Most of the studies shown in **Supplementary Table S2** did not have results with sustained inactivation; therefore, their claim about ant control was probably overestimated. In fact, those publications that had a length of four weeks or less should not be considered as valid, especially if only one application was made. In order to get a net effect of the experimental treatment with fungal inoculation, we have subtracted the percentage of mound inactivation of the control group from the percentage of the inoculated group (see **Supplementary Table S2**) and considered the net value in our criteria of mound inactivity. After doing this, we observed that a lower percentage of mound inactivation than reported was observed in 16 treatments coming from ten publications. Therefore, we can conclude that those studies had overestimated the efficiency of their control methods.

A small number of published studies evaluated the status of the nests at the end of the experiments (growing vegetation on *Atta* nests, or unmaintained foraging trails with abandoned holes covered with debris or sunken nests in *Acromyrmex*), with some reporting their observations as comments and not as quantitative data. The same happens with the few publications that claim to have excavated the inactive nests. This type of information is as valid as a sustained inactivity over time, and it is very important to quantify it, especially because most nests cannot be excavated due to their size or constraints imposed by landowners. In the latter case, if the nests are found to be empty, this observation should not be considered unequivocally as equivalent to colony death. In fact, it is known that many ants move their nests as part of their social defense strategy when they cannot deal with the infection (Oi and Pereira, 1993; Folgarait and Goffré, 2021b), or face unfavorable conditions (Guillade and Folgarait, 2018), or by unknown reasons (Oi et al., 1994; Bextine and Thorvilson, 2002). Therefore, the control efficacy will be overestimated if the colony moves prior to being considered dead.

Some low values (lower than 40%) of mound inactivation were registered in the studies reported here. Few studies use an estimation of mound size to decide how much product was needed to use for inoculation and, therefore, the amounts could have been insufficient in the other studies, producing an underestimation of the potential control efficiency. With the available data set it is very difficult to explain the possible reasons for those values. Probably, there are many more unpublished experiments with similar results, since journals do not normally accept manuscripts with “negative” results. Among our unpublished results, several times we have seen two types of ant behaviors that are worth considering in field experiments of ant control. On the one hand, we have seen ants from control groups “stealing” baits from the inoculated mounds. This behavior tends to produce an increase in mound inactivation in the control group, which is a result that does not help to correctly evaluate the control strategy (a situation that might be avoided if control nests are placed very far from nests treated with fungi). On the other hand, we have observed that most or all mounds (depending on the ant species) located in disturbed situations, such as plantations with LCA, do not exhibit aggression between each other, suggesting the existence of a high level of kinship and/or polydomy, characteristically found in exotic invasive species. If this is true, then the results obtained from control and inoculated groups will probably not be as predicted, and will be difficult to interpret. If native ants that become pests in their own land share many of the traits that characterize exotic/tramp species, then their control will be more difficult because the chance of eradicating a colony completely will diminish. In fact, all the field studies reported here for the invasive *S. invicta* (except one that had eight applications) showed a low net “mortality”, which suggests the difficulty in controlling this exotic species. Even for LCA, the most studied pest ant group in its native range, hardly nothing is known about those traits (only five LCA pest species were found to be polygyne; Delabie, 1989; Diehl-Fleig and Souza, 1999; Diehl et al., 2001).

### 4.3 Overall considerations

It is difficult to know exactly how many ant species are considered pests in the world (**Table 2**); however, 147 ant species have been recorded outside their native range (McGlynn, 1999) and, in general, exotic species become invaders if they establish, and soon afterwards become pests (Williams, 1994; Wetterer, 2009). In fact, the global invasive data set includes four species of ants (*Anoplolepis gracilipes*, *Linepithema humile*, *Solenopsis invicta*, and *Wasmannia auropunctata*), among the 100 world’s worst invasive alien species list (Lowe et al., 2000). In addition, 14 species of ants that invaded the Asian-Pacific areas are considered to be in the group of the 19 most destructive invader ant species (Xu et al., 2022). It is astonishing to realize that all the work done on the biological control of ants by fungi, which is one of the most ancient and studied strategies of biological control, is mainly concentrated in two groups, LCA and fire ants, although some other non-chemical strategies have been studied for a few of the other worst alien ant species (Wetterer and Porter, 2003; Wang et al., 2016). The concentration of information into the two groups mentioned may be more related to their economic direct impact (Xu et al., 2022) than to their spread throughout the continent/world (Suarez et al., 2001), and their negative impact on biodiversity (Holway et al., 2002). From our database, we found a few commercial products that were tested with ants, and none of them seemed specifically designed for ants except for the baits, and our own experience illustrates that it is more common to find unregistered than registered products in the market. The latter, in part, may be a consequence of the difficulty in their registration (Borgio and Sahayaraj, 2011), especially in developing nations. Therefore, there are many ant pest species that have not received considerable attention regarding their control despite being very conspicuous.

### 4.4 Recommendations

Laboratory experiments dealing with basic questions on ant-fungi interactions may give some insights that could help to better design field experiments and/or interpret some results. For example, it will be very valuable to evaluate the effect of ant venoms from different pest species on the germination capacity and fungal growth of entomopathogens. Also, the immunization of non-infected ants that obtain the fungal pathogens through social transfer should be studied to evaluate how common it is among pest ants. Previous examples can jeopardize the success of control strategies using fungal entomopathogens. In addition, it will be very useful to test the inoculation of conidia suspensions mixed with fungal toxins in order to evaluate if there could be a synergistic effect in controlling the ants. For LCA, testing the addition of antibiotics against actinomycetes in conidial suspensions might help to reduce the presence of these bacteria on *Acromyrmex* ants, making them more susceptible to the attack by fungal entomopathogens. Although most of the knowledge about ant-specific fungi comes from fungi that produce the so called “summit disease”, such as species from *Ophiocordyceps* (Ascomycota) and *Pandora* (Entomophtoromycota) (see references and Fungi section in De Bekker et al., 2018), there is only one report that deals with the possible use of a newly described Entomophthoralean species as a biological control agent against pest ants (Folgarait and Goffré, 2021b). Therefore, fungal pathogens, especially entomophthorales, are worth studying in depth due to their known capacity of producing epizootia and their specificity towards their hosts (Pell et al., 2001; Folgarait and Goffré, 2021b). More efforts need to be put into searching for them in nature, testing their efficiency in the laboratory, evaluating the possibility of their escalation, and comparing its control success to the use of generalist pathogens. In addition, we strongly recommend considering our discussion about the laboratory experiments for future laboratory assays.

This review shows that the biological control of pest ants by using entomopathogenic fungi is still in its infancy. Our main recommendation is to redirect the efforts towards field experiments, even if those are very expensive, logistically difficult, and should last a long time (and land owners may not accept or respect those times). Experiments should also have sample sizes of at least six to ten colonies/treatment, as well as always have a control treatment; in addition, it is advisable to do repeated applications and the observations discussed previously from field experiments should be considered and quantified. If logistics and number of colonies in the field allow, it will be very insightful if different doses can be tested. In addition, it will greatly help to know if applying the control at a particular time of the year and stage of the colony could increase the chances of a successful control. We hypothesize that controlling after a harsh season might be better because the ants may be bad nourished and more susceptible to acquire a disease. Similarly, the control of young colonies will be easier due to a smaller number of ants, but if it is necessary to deal with mature colonies, we propose to apply the product at the stage when the colony is investing most of its energy in producing alates, and therefore less energy will be available for defense.

It is important to understand the challenges of well replicated, long-lasting, field experiments. Each replicate (i.e. colony) includes hundreds to millions of individuals with one or many reproductive queens that have to be killed, and this is not comparable to solitary insect pests in which the death of a female represents one less individual that will reproduce and cause harm. If the queen from a colony is not killed during control, the colony will continue to grow despite initially appearing to be controlled when a reduction of workers is observed. The last point highlights previous statements about the need to increase the duration of field trials. Additionally, and in order to advance and not to repeat failures, “negative” results, should be submitted and accepted for publication for the sake of improving our knowledge and advancing at a faster pace in the biological control of ant pests.

## Data availability statement

The original contributions presented in the study are included in the article/**Supplementary Material**. Further inquiries can be directed to the corresponding author.

## Author contributions

PF and DG contributed to conception and design of the study. DG organized the laboratory database and PF the field one. PF performed the statistical analysis. PF wrote the first draft of the manuscript. All authors contributed to the article and approved the submitted version.
