# Supplementary material for: Control of pest ants by pathogenic fungi: state of the art
Source: Front Fungal Biol. 2023 Oct 11;4:1199110. doi: 10.3389/ffunb.2023.1199110 (PMC10598784; doi:10.3389/ffunb.2023.1199110)
Supplement: Supplementary file 2 [file Table_2.docx]

Supplementary Material

Control of pest ants by pathogenic fungi: state of the art

Patricia J. Folgarait ^*^, Daniela Goffré

*** Correspondence:**Corresponding Author
patricia.folgarait3@gmail.com, pfolgarait@unq.edu.ar

Table S2. Field experiments included in this study discriminated by ant species and fungal species used. Treatments with sample sizes are given for each experiment. Frequency, amount, and form of application of the fungi is specified. Mortality criteria, results, and net mound inactivity is also shown.

| Ant species | Fungal Strain (origin) | Experiment (N) | # applications | Frequency of Application & measurements | | Amount applied | | Concentration applied  (conidia/g or ml) | Form of application | | | Ant / Mound inactivity (%) | | | Net mound mortality (%) | Criteria mortality; comments on results (length of study) | | Reference (Country of the study) | |  |
| --- | --- | --- | --- | --- | --- | --- | --- | --- | --- | --- | --- | --- | --- | --- | --- | --- | --- | --- | --- | --- |
| *At. cephalotes* | *M. anisopliae*, M-137 &  *T. viride* T-26  (from same host) | Ma Site 1 (5) | 3 | 13^th^, 23^th^ & 36^th^ wk / every 10-13 wks | | 20 g/m^2^ | | 10^9^ (20% w/w) | Baits | | | 100% inactivity | | | 80 | W/o ant activity during 4 consecutive wks  Excavation of inactive nests at the end of the experiment  (42 wks) | | Lopez and Orduz (2003) (Colombia) | |  |
|  |  | Tv Site 1 (5) |  |  |  |  |  |  |  |  |  | 100% inactivity | | | 80 |  |  |  |  |  |
|  |  | Ma + Tv Site 1 (5) |  |  |  |  |  |  |  |  |  | 100% inactivity | | | 80 |  |  |  |  |  |
|  |  | Control Site 1 (5) |  |  |  |  |  | NA | Baits w/o fungi | | | 20% inactivity | | |  |  |  |  |  |  |
|  |  | Ma Site 2 (5) |  | 7^th^, 14^th^ & 19^th^ wk / Every 5-7 wks | |  |  | 10^9^ (50% w/w) | Baits | | | 100% inactivity | | | 100 |  |  |  |  |  |
|  |  | Tv Site 2 (5) |  |  |  |  |  |  |  |  |  | 60% inactivity | | | 60 |  |  |  |  |  |
|  |  | Ma + Tv Site 2 (5) |  |  |  |  |  |  |  |  |  | 100% inactivity | | | 100 |  |  |  |  |  |
|  |  | Control Site 2 (5) |  |  |  |  |  | NA | Baits w/o fungi | | | 0% inactivity | | |  |  |  |  |  |  |
|  | *B. bassiana*, ATCC MYA -4886  *T. lignorum*, ATCC-8751  (not indicated) | Bb (Mycotrol) Exp1 (3) | 3 | Every 4-5 wks / every wk | | 10 ml/m^2^ | | 10^9^ | Solution | | | Significant lower ant flow compared to control | | | NC | W/o ant activity, at least, at the end of experiment (14 wks) | | Fernández-Daza et al. (2019) (Colombia) | |  |
|  |  | Tl (Mycobac) Exp1 (3) |  |  |  |  |  | 10^9^ |  |  |  | Control increased its activity | | |  |  |  |  |  |  |
|  |  | Most virulent Bb + Tl Exp1 (3) |  |  |  |  |  | 10^9^ (1:1 ratio) |  |  |  | Most Effective Bb + Tl, least effective T1 | | |  |  |  |  |  |  |
|  |  | Control Exp1 (2) | ? | NA / every wk | | ? | | ? | ? | | | Results explained but data not shown | | |  |  |  |  |  |  |
|  | *Purpureocillium* sp, 0484  *Metarhizium* sp, RCP-2  *T. hammatum*, 0585  (collection; from non LCA hosts; except T isolated from same host) | P | 1 | NA / every wk | | 6 g/m^2^ | | 10^7^ (1%) | Baits | | | Proportional change in ant activity significantly greater than control (0.57); 20% mortality | | | 20 | Ant flow cero during 4 consecutive wks  (8 wks) | | Varón Devia (2006) | |  |
|  |  | M |  |  |  |  |  |  |  |  |  | Proportional change in ant activity not different from control; 10% mortality | | | 10 |  |  |  |  |  |
|  |  | Th |  |  |  |  |  |  |  |  |  | Proportional change in ant activity not different from control; 0% mortality | | | 0 |  |  |  |  |  |
|  |  | P + Th |  |  |  |  |  | 10^7^ (1:1 ratio) |  |  |  | Proportional change in ant activity not different from control; 20% mortality | | | 20 |  |  |  |  |  |
|  |  | Control | ? |  |  | ? | | NA | ? | | | Proportional change in ant activity (0.22). Mortality 0%. | | |  |  |  |  |  |  |
| *At. cephalotes* | *M. anisopliae*  *T. harzianum*  (collections) | M (4) | 3 | Every 2 wks / every wk | | 20 g/m^2^ | | 10^8^ | Insufflated powder | | | Significant reduction of activity (70%) compared to control; 50% of mound inactivation (small nests). Dead queens, M recovered from cadavers | | | 50 | No ant activity and nests empty; with compacted soil and plants growing on it.  (6 wks) | | Barrera Llano (2006) (Colombia) | |  |
|  |  | T (4) |  |  |  |  |  | 10^9^ |  |  |  | Reduction of ant activity (26%) not different from control. Mound inactivation of 0% | | | 0 |  |  |  |  |  |
|  |  | M + T (4) |  |  |  |  |  | 10^8^ + 10^9^ |  |  |  | Significant reduction of activity (60%) in comparison to control; 50% of mound inactivation (small nests) | | | 50 |  |  |  |  |  |
|  |  | Control (4) | 0 | NA/ every wk | | NA | | NA | NA | | | Increased activity by 55%. Mortality 0% | | |  |  |  |  |  |  |
| *At. colombica* | *B. bassiana* BAZAM  *T. harzianum* TRICHOZAM | Bb (Mycotrol) Exp1 (4) | 2 | Every 2 wks / 3 times per wk | | 150 g (small nests) / 250 g (big) | | 10^11^ (8%) | Insufflated powder | | | Significant reduction of ant activity | | | NC | Ant activity from all holes  (4 wks) | | Banderas (2004) (Honduras) | |  |
|  |  | Th (4) |  |  |  |  |  |  |  |  |  | Significant reduction of ant activity | | |  |  |  |  |  |  |
|  |  | Control (4) | 0 | NA / 3 times per wk | | NA | | NA | NA | | | Increased ant activity through time | | |  |  |  |  |  |  |
| *Atta* sp. | *B. bassiana*  *M. anisopliae*  (from LCA species) | Bb (4) | 2 | | 7 & 14 d / every other day | 20 L | 10^12^ UFC/ml (0.2 L for small nests / 0.4 L for big) | | | | spray pump | 3% of traffic reduction, significantly different to control | | | NC | Reduction of ant activity, considering initial traffic 100%  (30 d) | | Hernandez (2016) (Guatemala) | |  |
|  |  | Ma (4) |  |  |  |  |  |  |  |  |  | 33% of traffic reduction, significantly different to control | | |  |  |  |  |  |  |
|  |  | CT | NA | NA / every other day | | NA | NA | | | | NA | 52% of traffic increase at day 14 | | |  |  |  |  |  |  |
| *Ac. heyeri* + *Ac.crassispinus* | *B. bassiana* BSA  (from another LCA host) | Bb (49) | 1, 2, or 3 | every 7 d / 14, 21, 35 d | | 40 g | | 10^10^ | Inoculated over the symbiotic fungi | | | 87% of inactive nests | | | 87 | Nest inactivity  (35 d) | | Diehl-Fleig et al. (1993) (Brazil) | |  |
|  |  | Control (13) | ? | ? / 14, 21, 35 d | | ? | | NA | NA | | | 0% inactive nests | | |  |  |  |  |  |  |
| *Acromyrmex* spp.* | *B.bassiana* BSA  (from another LCA host) | Bb (148) | 1 | NA / 7, 14,21, 35 d | | 40 g | | 10^10^ | Inoculated over the symbiotic fungi | | | 83% of inactive nests | | | 83 | Nest inactivity  (35 d) | | Diehl-Fleig et al. (1993) (Brazil) | |  |
|  |  | control (44) | ? |  |  | ? | | NA | NA | | | 0% of inactive nests | | |  |  |  |  |  |  |
| *Ac. heyeri* | *M. anisopliae* MVHC 1878  (from soil) | Ma T1/T2/T5/T6 (5/T) | 3 | every 7 d / every wk | | 1L (T1&T2) 2L (T5 & T6) | | 10^7^ | Insufflation after disturbance | | | 60% inactive nests (small); 100% inactive nests (big); both significantly different to controls | | | 70 | Nest inactivity  (4 weeks) | | Tiscornia et al. (2014) (Uruguay) | |  |
|  |  | Control (5) | ? | NA / every wk | | ? | | NA | ? | | | 0 to 20% inactive nests (not discriminated by size) | | |  |  |  |  |  |  |
|  |  | Ma T3 (5) | 3 | every 7 d / every wk | | 1L | | 10^8^ | Insufflation after disturbance | | | 60% inactive nests; not significantly different to control | | | 60 |  |  |  |  |  |
|  |  | Control (5) | ? | NA / every wk | | ? | | NA | ? | | | 0% inactive nests | | |  |  |  |  |  |  |
|  |  | Ma T4 (5) | 3 | every 7 d / every wk | | 2L | | 10^10^ | Insufflation after disturbance | | | 100% inactive nests; significantly different to control | | | 80 |  |  |  |  |  |
|  |  | Control (5) | ? | NA / every wk | | ? | | NA | ? | | | 20% inactive nests | | |  |  |  |  |  |  |
| *Ac. landolti*  *fracticornis* | *B. bassiana*  BOVENAT  *M. anisopliae* METANAT | Bb (10) | 1 | NA / 8, 15, 22, 30, 60 d | | 20 g | | 10^10^ | Insufflation | | | 80% efficiency | | | 80 | Product efficiency based on active & inactive nests in inoculated and control treatments at the end  Excavation of dead nests (60 d) | | Amarilla Salinas and Arias Ruiz Díaz (2011) (Paraguay) | |  |
|  |  | Ma (10) |  |  |  |  |  | 10^10^ |  |  |  | 70% efficiency | | | 70 |  |  |  |  |  |
|  |  | Bb + Ma (10) |  |  |  |  |  | 50% of each fungus |  |  |  | 70% efficiency | | | 70 |  |  |  |  |  |
|  |  | Control | ? |  |  | ? | | NA | ? | | | Mentioned was done but no data reported | | |  |  |  |  |  |  |
| *Ac. lundii* | *M. anisopliae* MVHC 1878  (from soil) | Ma T9/T10 (5/T) | 3 | every 7 d / every wk | | 10 g | | 10^8^ | Insufflation in holes | | | 60% inactive nests in T9 and 40% in T10; not significantly different to control | | | 60 (T9)  20 (T10) | Nest inactivity  (4 wks) | | Tiscornia et al. (2014) (Uruguay) | |  |
|  |  | Control (5) | ? | NA / every wk | | ? | | NA | NA | | | 0% inactive nests in T9 and 20% in T10 | | |  |  |  |  |  |  |
|  |  | Ma T7/T8 | 3 | every 7 d / every wk | | 30 g | | 10^10^ | Insufflation in holes | | | 100% inactive nests in T7 & T8; significantly different to control | | | 100 |  |  |  |  |  |
|  |  | Control (5) | ? | NA / every wk | | ? | | NA | NA | | | 0% inactive nests in T7 & T8 | | |  |  |  |  |  |  |
| *Ac. lundii* | *B. bassiana* (B5/B6/B7)  *T. lentiforme* (T1/T2/T3) *M. anisopliae* (MFPN twice/MFAV) (different LCA species) | Bb + Tl (11) | 12 (4 kits with 3 baits each kit) | 1 bait per wk. Kits every 1-2wks except de third after 3 months (winter) / every wk | | 50 g/trail; all trails | | B: 10^7^ T: 10^6^ | Baits | | | 73% of inactive nests at 44th wk | | | 59 | Mound sustained inactivation for 13 wks; holes and trails abandoned  (31 wks) | | Folgarait (2019) (Argentina) | |  |
|  |  | Bb + Tl + Ma (6) |  |  |  |  |  | B:10^7^T:10^6^ M:10^6^ |  |  |  | 67% of inactive nests | | | 53 |  |  |  |  |  |
|  |  | Control (7) |  |  |  |  |  |  | Baits w/o fungi | | | 14% of inactive nests | | |  |  |  |  |  |  |
|  | *B. bassiana* (B6)  *T. virens* (T4) (different LCA species) | Bb + Tv (8) | 9 (3 kits with 3 baits each kit) | Baits every 1/2wk. Between 1st and 2nd kits: 9 wks; between 2nd and 3rd: 5 wks/every wk | | 40 g/trail in 3 trails | | B: 10^7^ T: 10^6^ to 10^8^ | Baits | | | 87,5% after 27 wks when last bait offered but 62,5% at 45, and 54 wks | | | 63 | Mound sustained inactivation for 13 wks; holes and trails abandoned  (27 wks) | | Folgarait and Goffré (2021a) (Argentina) | |  |
|  |  | Control (7) |  |  |  |  |  |  | Baits w/o fungi | | | 0 % of inactive nests | | |  |  |  |  |  |  |
| *Ac. octospinosus* | *B. bassiana*  BIBISAV-2 | Bb (4) | 1 | NA/ 7,15,30,60,90d | | 100 g /m^2^ | | 10^9^ | ? | | | 100% efficiency at 60 days, maintained at 90d; cero holes, 0 area | | | 100 | - Product efficiency based on active & inactive nests in inoculated and control treatments  - Number of holes, evaluation of nest area  (7.5 wks) | | Álvarez and González (2002) (Cuba) | |  |
|  |  | Control (4) | 0 |  |  | NA | | NA | NA | | | Increases area & number of holes | | |  |  |  |  |  |  |
| *S. invicta* | *B. bassiana* BB2484  (from *At. mexicana* re-isolated afterwards from fire ants) | Bb (insertion of pellets in mound) (50) | 1 | NA / 8, 31, 51d | | 2 g | | ? | Bb mycelia alginate pellets | | | Mean mound rate not significantly different from control at any sampling date | | | NC | Ratings based on ant numbers and brood presence when surface mound scrapped  (7 wks) | | Bextine and Thorvilson (2002) (USA) | |  |
|  |  | Control w/o fungi (50) |  |  |  |  |  | NA | Alginate paper pellets | | | Mean mound rate not significantly different from control at any date | | | NC |  |  |  |  |  |
|  |  | Control w/o pellets (50) | NA |  |  | NA | | NA | NA | | |  | | |  |  |  |  |  |  |
|  |  | Bb (broadcast application 1997) (25) | 1 | NA / 15, 30, 52, 91d | | 2 g | | ? | Bb alginate pellets with oil coating | | | Significantly lower rates than control from d19 on; 72% mounds inactive; 32% cadavers with Bb | | | 51 | Ratings based on ant numbers and brood presence when surface mound scrapped  (13 wks) | |  |  |  |
|  |  | Control w/ Bb w/o coating pellets (22) |  |  |  |  |  | ? | Alginate pellets wo coating | | | No differences with the control for any date; 32% mounds inactive; no cadavers with Bb | | | 11 |  |  |  |  |  |
|  |  | Control w/o pellets (33) | NA |  |  | NA | | NA | NA | | | 21% mounds inactive; no cadavers with Bb | | |  |  |  |  |  |  |
| *S. invicta* | *B. bassiana* BB2484  (from *At. mexicana* re-isolated afterwards from fire ants) | Bb (broadcast application 1998) (59) | 1 | NA / every 2 wks | | 12 g /ha | | ? | Bb alginate pellets with oil coating | | | Significantly lower rates from d14 on; 90% mounds inactive; 45% cadavers with Bb | | | >90 | Ratings based on ant numbers and brood presence when surface mound scrapped  (12 wks) | | Bextine and Thorvilson (2002) (USA) | |  |
|  |  | Control w/ Bb w/o coating pellets (52) |  |  |  | ? | | ? | Bb alginate pellets w/o coating | | | No differences with the control for any date; 35% more mounds active; no cadavers with Bb | | | >35 |  |  |  |  |  |
|  |  | Control w/o pellets (60) | NA |  |  | NA | | NA | NA | | | 18% more mounds active; no cadavers with Bb | | |  |  |  |  |  |  |
|  |  | Bb (pellets with oil coating on the ground) (15) | 1 | NA / 14, 28, 42, 56 d | | 20 pellets | | ? | Bb alginate pellets with oil coating | | | Significantly lower rates than control since 8d; 80% mounds inactive; 100% cadavers with Bb | | | 40 | Ratings based on ant numbers and brood presence when surface mound scrapped  (8 wks) | |  |  |  |
|  |  | Control w/ Bb w/o coating pellets (15) |  |  |  | NA | | NA | Bb alginate pellets w/o coating | | | Sign. lower rates than control since 51d; 40% mounds inactive; 0% cadavers with Bb | | | 13 |  |  |  |  |  |
|  |  | Control w/o pellets (15) | NA |  |  | NA | | NA | NA | | | 27% mounds inactive; no cadavers with Bb | | |  |  |  |  |  |  |
|  |  | Bb (pellets with oil coating on the ground) (208) | 1 | NA / every other wk | | 2 g | | ? | Bb alginate pellets with oil coating | | | Sign. lower rates than control since 14d; 54% mounds inactive; 0% cadavers with Bb | | | NC | Ratings based on ant numbers and brood presence when surface mound scrapped  (12 wks) | |  |  |  |
|  |  | Control (paper pellets coated with oil) (203) | NA |  |  | NA | | NA | Alginate paper pellets with coating | | | No changes in ant ratings through time, inactive mounds not reported | | |  |  |  |  |  |  |
|  | *B. bassiana* 447  (from native *S. invicta*) | Bb (surface application) (50) | 1 | NA / 1, 4, 8 wks | | 200 g | | 5% of fungus | | Rice dispersed on disturbed mounds | | | 62% mounds inactive at the end, and 32% new mounds | 36 | | | Active if >30 ants were present after disturbance; inactive when less > 30 came out; baited traps for quantification of foraging ants  (8 wks) | | Oi et al. (1994) (USA) | |
|  |  | Control (50) |  |  |  | 200g | | NA | |  |  |  | 26% mounds inactive and 18% new mounds |  | | |  |  |  |  |
|  |  | Bb injected w/carrier1 injected w/ CO_2_ (10) | 1 | NA / 1, 4, 8 wks | | 100 g | | 10% of fungus | | Injection using probes | | | 75% mounds inactive, 75 % of new mounds, significant lower number of ants and reduction of ants on traps | 25 | | |  |  |  |  |
|  |  | Control injected with compressed air (6) |  |  |  | 100 g | | NA | |  |  |  | 50% mounds inactive, 75% new mounds |  | | |  |  |  |  |
|  |  | Bb + carrier2 injected with CO_2_ (41) | 1 | NA / wk 1 & 4 | | 7 to 50 g/mound | | 10% of fungus | | Injection using probes | | | 100% mounds inactive at 4wk, 34% of new mounds | 6 | | |  |  |  |  |
|  |  | Control w/carrier2 injected w/ CO_2_ (34) |  |  |  | 3 to 50 g/mound | | NA | |  |  |  | 94% mounds inactive and 35% new mounds, significant lower number of ants | 13 | | |  |  |  |  |
|  |  | control injected w/ CO_2_ (37) |  |  |  | NA | | NA | NA | | | 81% mounds inactive, 51% of new mounds, significant lower number of ants but equal to the Bb treatment | | |  |  | |  | |  |
| *S. invicta* | *B. bassiana* ZGNKY-5  (another host insect) | Bb T1 (3) | 1 | NA / 4, 10, 20, 30 d | | 100 ml | | 10^8^ | Injection with a 3-probe with spray injector | | | Nest rates did not decrease significantly different than the control but there was significant reduction of ant activity | | | NC | Ant activity with baited traps around the mound. Mound active if >3ants emerged within 60s after poking the mound with a stick.  (4 wks) | | Li et al. (2016) (China) | |  |
|  |  | Bb T2 (3) |  |  |  | 250 ml | |  |  |  |  | Nest rates did not decrease significantly different than the control but there was significant reduction of ant activity | | |  |  |  |  |  |  |
|  |  | Bb T3 (3) |  |  |  | 500 ml | |  |  |  |  | Nest rates decreased significantly and there was significant reduction of ant activity, both in comparison to control. | | |  |  |  |  |  |  |
|  |  | Bb T4 (3) |  |  |  | 750 ml | |  |  |  |  | Nest rates decreased significantly and there was significant reduction of ant activity, both in comparison to control. | | |  |  |  |  |  |  |
|  |  | Bb T5 (3) |  |  |  | 1000 ml | |  |  |  |  | Nest rates decreased significantly and there was significant reduction of ant activity, both in comparison to control. | | |  |  |  |  |  |  |
|  |  | Control (Tween 80+H2O) (3) |  |  |  | 1000 ml | | 0.05% (v/v) |  |  |  |  | | |  |  |  |  |  |  |
|  | *B. bassiana* F256  (unknown insect) | Bb baits dispersed (11) | 1 | NA / 7,14,21,28 d | | 60 ml | | 10^7^ | Baits | | | Significantly greater number of inactivated mounds (27.3%) than the control. | | | 18 | Number of active mounds (when 20 workers exited the mound after disturbed with a rod).  (4 weeks) | | Kafle et al. (2011) (Taiwan) | |  |
|  |  | Bb liquid injected (11) |  |  |  |  |  |  | Liquid injected within the mound | | | Significantly greater number of inactivated mounds (54.5%) than the control and the B-bait. | | | 46 |  |  |  |  |  |
|  |  | Control (11) | ? |  |  | ? | | ? | ? | | | 9% of inactivated mounds | | |  |  |  |  |  |  |
|  | *B. bassiana* N18  (another host insect) | Bb (35) | 8 | every wk 1st mth, every 2 wks 2nd and 3rd mth / ? | | 5 g | | ? | Formulation inserted in holes | | | 100% inactive mounds wo living ants or brood | | | 95 | Monitoring of ant activity (not explained how) and excavation of inactive mounds  (12 wks) | | Rojas et al. (2018) (USA) | |  |
|  |  | Control (15) |  |  |  | NA | | NA | NA | | | 5% of inactive nests (but chemically sprayed), the remaining were well established. | | |  |  |  |  |  |  |
| *S. geminata* | *B. bassiana*  *M. anisopliae*  (from commercial strain) | Bb (10) | 1 | NA / every day | | 100 g | | 3, 5, 7, and 10 g+ | Formulation scattered over a disturbed mound | | | 100% of nests inactive at all treatments, except in treatment with 3g | | | 100 | Mound inactivation  (5 days) | | Nalini and Sasinathan (2020) (India) | |  |
|  |  | Control (10) | NA |  |  | NA | | NA | NA | | | no inactive mounds | | |  |  |  |  |  |  |
|  |  | Ma (10) | 1 | NA/ every day | | 100 g | | 3, 5, 7, and 10 g + | Formulation scattered over a disturbed mound | | | 0, 10, 20, and 40% mounds were inactive, for each treatment respectively. | | | 17.5 |  |  |  |  |  |
|  |  | Control (10) | NA |  |  | NA | | NA | NA | | | no inactive mounds | | |  |  |  |  |  |  |
| *S. saevissima* | *B. bassiana* strain 447  (from same host) | Bb (3/T) | 1 | NA/ at 28, 87, and 100 d | | 12.5, 25, 50, 100, or 200 g | | 10^15^ | Formulation inserted in the mound | | | 100% of inactive and dead nests for the 12.5 and 100 UFC/g treatments, the others 66.7% | | | 82.5 | Inactive mounds if no ants emerged after 60 s of disturbance, and excavation of inactive nests  (12 weeks) | | Stimac et al. (1989) (Brazil) | |  |
|  |  | Control (5) |  |  |  | 0 | | NA | NA | | | no inactive mounds | | |  |  |  |  |  |  |
| *Lasius niger* | *Penicillium* sp  (organic oranges) | Orange peel with *Penicillium* (3/T) | 3 | every day / every day | | 25, 50 & 100% (v/v) | | 100%: 10^6^ | Spray on foraging ants | | | No significant differences among treatments or concentrations | | | NC | Percentage of dead ants sprayed on foraging trails  (3 d) | | Dávila Pino et al. (2018) (Ecuador) | |  |
|  |  | Orange peel without fungi (3/T) |  |  |  |  |  |  |  |  |  |  |  |  |  |  |  |  |  |  |
|  |  | Only fungi (3/T) |  |  |  |  |  |  |  |  |  |  |  |  |  |  |  |  |  |  |
|  |  | Positive control (not specified) (3) | ? |  |  | ? | | ? | ? | | | High mortality | | |  |  |  |  |  |  |
|  |  | Negative control (not specified) (3) | ? |  |  | ? | | ? | ? | | |  | | |  |  |  |  |  |  |

Footnote: Abbreviations used in the table: N = number of replicates; NA = not applicable; NC = mound inactivity cannot be calculated; ? data not shown in the publication; +: concentration not indicated

*: includes: *A. aspersus, A. crassispinus, A. lundii, A. subterraneus bruneus*
